# Multi-omics data-based modeling reveals tumorigenesis- and prognosis-associated genes with clinical potential in lung adenocarcinoma

**DOI:** 10.1186/s12885-025-14943-x

**Published:** 2025-11-10

**Authors:** Zhendong Lu, Pengfei Bao, Taiwei Wang, Kairui Hu, Lina Zhang, Ling Yi, Yuanming Pan, Weiying Li, Zhi John Lu, Jinghui Wang, Junzhong Ruan

**Affiliations:** 1 Department of Medical Oncology, Beijing Chest Hospital, Capital Medical University/Beijing Tuberculosis and Thoracic Tumor Research Institute, Beijing, 101149 China; 2 MOE Key Laboratory of Bioinformatics, Center for Synthetic and Systems Biology, School of Life Sciences, Tsinghua University, Beijing, 100084 China; 3 Institute for Precision Medicine, Tsinghua University, Beijing, 100084 China; 4 Joint Graduate Program, School of Life Sciences, Peking University–Tsinghua University–National Institute of Biological Sciences, Tsinghua University, Beijing, 100084 China; 5 Academy for Advanced Interdisciplinary Studies (AAIS), and Peking University, National Institute of Biological Sciences Joint Graduate Program (PTN), Tsinghua University, Peking University, Beijing, 102206 China; 6 Cancer Research Center, Beijing Chest Hospital, Capital Medical University/Beijing Tuberculosis and Thoracic Tumor Research Institute, Beijing, 101149 China; 7 Department of Thoracic Surgery, Beijing Chest Hospital, Capital Medical University/Beijing Tuberculosis and Thoracic Tumor Research Institute, Beijing, 101149 China

**Keywords:** Deep learning, ATAC-seq, LUAD, Tumorigenesis prognosis, Early cancer detection, ScRNA-seq, Multiomics

## Abstract

This study aims to utilize multi-omics high-throughput sequencing data, including ATAC-seq and RNA-seq data from TCGA, GTEx, and GEO databases, to construct predictive and prognostic models for lung adenocarcinoma (LUAD) and identify potential biomarkers. We first obtained LUAD ATAC-seq data from TCGA and identified differential chromatin regions and genes through functional analysis. Differential peaks (DPs) potentially influencing LUAD progression were determined by analyzing patients at different stages, and these DPs were annotated to the genome to obtain differential peak genes (DPGs). We then integrated RNA-seq data from GTEx and TCGA to identify differentially expressed genes (DEGs) at the mRNA level, and by intersecting DEGs with DPGs, we identified 337 consensus genes (CGs). Using random forest and LASSO algorithms, we screened the CGs and constructed a predictive model comprising nine predictive-related genes (Pre-RGs), which was validated with an external dataset (GSE140343). Additionally, through Kaplan-Meier and Cox analyses combined with LASSO, five prognostic-related genes (Pro-RGs) were identified and used to establish a prognostic Cox proportional hazards model, also validated by GSE140343. Single-cell dataset analysis examined the expression of Pre-RGs and Pro-RGs across immune cell types, and further meta-analysis in the LCE database verified their expression differences and prognostic significance. Furthermore, we sequenced cell-free RNAs (cfRNAs) from 50 plasma samples (25 early-stage lung cancer and 25 benign pulmonary disease cases) to validate early cancer detection. Overall, we identified signatures including *S100A8*, *GPM6A*, *FEZ1*, *OTX1*, *DNAH14*, *XDH*, *XPR1*, *SLC39A11*, *OCIAD2*, *TNS4*, *RHOV*, *YWHAZ*, *CLEC12A*, and *CASZ1*, which show potential as drug targets and biomarkers for predicting LUAD development, prognosis, and early detection.

**Supplementary Information:**

The online version contains supplementary material available at 10.1186/s12885-025-14943-x.

## Introduction

Lung adenocarcinoma (LUAD) is a malignant tumor originating from the glandular epithelium of the bronchial mucosa and classified as a type of non-small cell lung cancer (NSCLC) [1, 2]. LUAD tends to occur in the peripheral regions of the lungs and is more prevalent in women. Characterized by rich vascularity, LUAD facilitates early hematogenous metastasis and is associated with poor prognosis [3]. At diagnosis, over half of LUAD patients present with local or distant metastases, resulting in a median survival of only 10 months [4–6]. Despite advancements in surgical, chemotherapeutic, radiotherapeutic, targeted, and immunotherapeutic treatments, the overall 5-year survival rate for LUAD patients remains as low as 25% [7]. These statistics underscore the critical importance of early diagnosis and identify prognostic biomarkers and develop more effective therapeutic strategies for LUAD patients.

Assay for Transposase-Accessible Chromatin using sequencing (ATAC-seq) is a high-throughput sequencing technique that rapidly and reliably identifies open chromatin regions in the genome [8–10]. These accessible regions play crucial roles in gene expression regulation. By analyzing their dynamic changes, we can gain a better understanding of the regulatory mechanisms involved in LUAD pathogenesis and progression [11, 12]. ATAC-seq allows for the detection of chromatin accessibility at specific gene loci, revealing their regulatory relationships with transcription factors [13, 14]. Using this approach, we can delve deeper into the molecular mechanisms and individual heterogeneity of LUAD, potentially identifying new prognostic genes.

In this study, we aim to perform an integrated analysis of ATAC-seq data with The Cancer Genome Atlas (TCGA) [15], Genotype-Tissue Expression (GTEx) [16], and transcriptomic data such as GSE140343. The advantage of this method lies in its ability to utilize complementary information to reveal the molecular characteristics of LUAD from multiple perspectives. ATAC-seq identifies open chromatin regions in the genome, while transcriptomic data provide insights into gene expression levels. By synergizing these datasets, we can achieve a more comprehensive understanding of the mechanisms regulating LUAD, potentially uncovering novel therapeutic targets and prognostic markers to support personalized treatment strategies. (The flowchart of the study is outlined in File S1, Figure S1)

## Methods

### Data sources

The ATAC-seq data for LUAD were sourced from The Cancer Genome Atlas (TCGA) database (https://gdc.cancer.gov/about-data/publications/ATACseq-AWG). This dataset includes a total of 22 LUAD samples. Data extraction was performed by accessing the “normalized counts from all cancer types-specific count matrices” file, and sample ID conversion was done using the “lookup table for various TCGA sample identifiers.” The corresponding ATAC-seq data for common sample names were obtained and standardized using R software. LUAD RNA-seq data, including fragments per kilobase per million mapped fragments (FPKM) and clinical sample information, were downloaded from the University of California, Santa Cruz (UCSC) database. This dataset comprises 526 tumor samples (reduced to 515 after removing duplicates), 59 normal samples, and 288 normal samples from the GTEx data pertaining to lung tissue. Additionally, RNA-seq data from GSE140343 were obtained from the NCBI Gene Expression Omnibus (GEO; https://www.ncbi.nlm.nih.gov/geo/), encompassing 51 LUAD tumor tissues and 49 adjacent non-cancerous tissues. Single-cell data from GSE131907 were also analyzed, selecting 11 in situ lung cancer samples for further examination.

### Chromatin accessibility analysis of LUAD ATAC-seq data

To explore chromatin accessibility in LUAD, we first visualized the chromatin coverage of ATAC-seq peaks using the “karyoploteR” package in R. Aligned peaks were mapped to TSS (Transcription Start Sites) regions to construct a tag matrix using the “ChIPseeker” package in R. Annotation of peak regions near TSS was performed with the “TxDb.Hsapiens.UCSC.hg38.knownGene”, “org.Hs.eg.db”, and “clusterProfiler” packages. We generated heatmaps to reveal the relationship between open chromatin regions and promoter areas. Additionally, we conducted Gene Ontology (GO) functional and Kyoto Encyclopedia of Genes and Genomes (KEGG) pathway enrichment analysis of genes annotated from all LUAD ATAC-seq data peaks using “clusterProfiler” and “ggplot2” packages, and visualized the results.

### Differential expression analysis using RNA-seq

To assess differential mRNA expression, the “Limma” package (version 4.2.2) in R was used. Adjusted *P*-values from the combined TCGA and GTEx datasets were analyzed to correct for false positives. Differentially expressed genes (DEGs) between normal and LUAD samples were identified using the Wilcoxon rank-sum test. DEGs were filtered based on the criteria of log2|FC (fold change) | >1 and FDR < 0.05. Heatmaps and volcano plots were created for visualization using the “ggplot2” package in R.

### Analysis of differentially open peaks in LUAD ATAC-seq between stage I VS II-IV

LUAD ATAC-seq peaks were divided into two groups based on TNM staging: Stage I (10 samples) and Stages II-IV (12 samples). Using R software, the “DESeq2” package was employed to perform differential peak analysis with a negative binomial distribution model. Peaks were filtered with the criteria log2|FC| >1 and *P* < 0.05. Differential peaks were annotated to promoter regions using the “ChIPseeker”, “TxDb.Hsapiens.UCSC.hg38.knownGene”, “org.Hs.eg.db”, and “clusterProfiler” packages to identify differential peak genes (DPGs).

Common genes (CGs) were identified by intersecting the DEGs and DPGs. Subsequent KEGG and GO analyses were performed using the “clusterProfiler” package in R to investigate the most significantly enriched pathways and biological processes of the CGs.

### Random forest and LASSO analysis for predictive gene selection

After obtaining the DEGs and DPGs, their intersection yielded CGs. A random forest model was created using the “randomForest” package in R. The optimal number of variables at each binary tree node was set to 6, and the total number of trees in the forest was set to 500. The algorithm calculated the mean decrease in accuracy for all variables between the LUAD and normal groups in the TCGA data. The optimal number of trees was determined by minimizing the cross-validation error rate. For subsequent modeling, the top 40 genes with the highest importance rankings were selected as representative genes, following a sample-to-parameter ratio of approximately 20:1 for over 800 samples [17].

Simultaneously, a LASSO model was constructed using the “glmnet” package [18]. In the LASSO regression coefficient trajectory plot, each curve represents the trajectory of a variable’s coefficient. By using the sum of the absolute values of the regression coefficients as the penalty function, features with minimal correlation to the predictive outcome were shrunk to zero, achieving variable selection. The intersection of genes identified by both the random forest and LASSO models yielded predictive related genes (Pre-RGs), which were used for constructing the subsequent artificial neural network (ANN) predictive model.

### Construction and validation of the ANN predictive model

Expression data for the Pre-RGs were analyzed to obtain Pre-RG gene scores. First, genes were divided into two groups based on their direction of differential expression (upregulated or downregulated). For each group, the median expression value was calculated as a threshold, and gene expression data were binarized. For the upregulated gene group, expression values greater than the median were labeled as 1, otherwise as 0; for the downregulated gene group, expression values less than the median were labeled as 1, otherwise as 0. This process resulted in a Pre-RG scoring matrix. An ANN model was constructed using the “neuralnet” package in R, which included five hidden layers. The fundamental principle of this model is that the sum of the weighted input gene scores determines the output layer. The “NeuralNetTools” package was used for visualizing the neural network model. The “pROC” package was employed to plot the ROC curve and calculate the area under the curve (AUC) to evaluate the performance of the neural network model.

For external validation, GSE140343 RNA-seq data were used. Pre-RG scores were calculated for the validation set using the same method. The gene scores from the validation set were input into the model to predict the scores for the test samples, thus determining the grouping of the validation set and calculating the prediction accuracy for LUAD and normal groups. The ROC curve was then plotted, and the AUC was calculated.

### Construction of the LUAD prognostic model

First, the “survival” package in R was used to perform univariate Cox regression analysis on the CGs with a significance threshold of *P* < 0.01, identifying significant variables. Next, Kaplan-Meier survival curves were plotted using the “ggsurvplot” package with a threshold of *P* < 0.05 to further filter variables. Subsequently, the “glmnet” package was used to apply Lasso regression for variable selection and dimensionality reduction. The penalty parameter (λ) of the Lasso model was selected based on the minimum criteria using 10-fold cross-validation.

Further screening of genes was performed using stepwise regression (with the parameter direction = “both”), constructing a Cox proportional hazards model to obtain the final prognostic related genes (Pro-RGs). The model predicts the risk score for each patient, calculated as follows: $\:risk\:score\:=\:{\sum\:}_{i=1}^{n}{coef}_{i}\:\times\:\:{Exp}_{i}\:$, where “*coef*” represents the coefficient and “*Exp*” represents the expression level of each Pro-RG. Patients were divided into high-risk and low-risk groups based on the median risk score. The “pheatmap” package was used to plot the risk status, survival state, and expression heatmap for the patients.

The “survminer” package was used to perform log-rank tests to obtain the *P*-value for survival differences between the two groups. Hazard ratios with 95% confidence intervals were obtained through univariate Cox regression analysis, and Kaplan-Meier plots were generated. The “timeROC” package was employed for 2-year, 3-year, and 4-year receiver operating characteristic (ROC) analysis, and the AUC values were calculated.

To validate the model’s accuracy, GSE140343 RNA-seq data were used as an external validation set. Risk status, survival state plots, expression heatmaps, and ROC curves were generated for the validation set. The “survival” and “rms” packages were used to plot calibration curves for the TCGA and GSE140343 datasets and to calculate the concordance index (C-index) of the prognostic model. Calibration curves assess the accuracy of the predictive model by comparing predicted probabilities to observed probabilities, while the C-index evaluates the model’s discriminative ability, ranging from 0.5 to 1.0, with higher values indicating better predictive performance.

Additionally, clinical characteristics of the entire TCGA LUAD cohort, including age, gender, and stage, were obtained. Using the “survival” package, univariate and multivariate Cox regression analyses incorporating the prognostic gene scores were conducted to independently validate the model. Visualization was performed using the “forest” R package. The “rms” and “regplot” R packages were used to establish a nomogram based on TCGA data, integrating age, gender, pathological stage, T, N clinical characteristics, and prognostic gene scores to assess the predictive accuracy of 2-year, 3-year, and 4-year survival probabilities.

Correlation of LUAD Prognostic Gene Scores with Immune Infiltration and Cancer Stemness Index.

To identify gene sets with statistically significant differences between high-risk and low-risk groups, Gene Set Enrichment Analysis (GSEA) was conducted. Gene sets annotated in the MSigDB database, including “c2.cp.kegg_legacy.v2023.2.Hs.symbols.gmt” and “c5.go.v2023.2.Hs.symbols.gmt” from KEGG and GO pathways, were analyzed. Enrichment of gene sets with adjusted *P*-values < 0.05 was considered significant. The R package “ESTIMATE” was used to assess Tumor Microenvironment (TME) scores (stromal score, immune score, and estimate score) between high-risk and low-risk groups to understand the proportions and changes of stromal and immune components in the LUAD tumor microenvironment.

Immune cell infiltration data for all TCGA tumors were downloaded from the TIMER2.0 online analysis platform (http://timer.comp-genomics.org/timer/) [19]. Spearman correlation analysis was performed to investigate the correlation between risk scores and immune cell infiltration in the high-risk and low-risk groups. The results were visualized using the “ggplot2” package in the form of a bubble plot. Additionally, the “GSVA” package was used to perform differential analysis of immune cells and immune-related functions between high-risk and low-risk groups using single-sample Gene Set Enrichment Analysis (ssGSEA), and the results were visualized using boxplots. Furthermore, LUAD stemness characteristics were evaluated using “StemnessScores_RNAexp” data obtained from previous studies [20].

### The correlation between prognostic gene scores in LUAD and TMB and ICGs

Using the “maftools” R package, mutation annotation format (MAF) data was extracted from the TCGA database to assess mutations across different prognostic gene score groups in LUAD patients. Total Mutation Burden (TMB) was calculated for each patient to quantify tumor mutation load, and differences in TMB between groups with different prognostic gene scores were compared. TMB serves as an indicator of the number of mutations in a tumor, often correlating with improved response to immune therapies. Additionally, the “surv_cutpoint” function from the “survminer” package was employed to determine the optimal cutoff for TMB scores, dividing patients into high and low TMB groups for prognostic analysis. Furthermore, a set of 79 Immune Checkpoint Genes (ICGs) was obtained from the literature [21], and differential analysis was conducted between high-risk and low-risk groups defined by prognostic gene scores. The correlation between ICGs and both prognostic gene scores and genes in the prognostic model was assessed.

### The correlation between LUAD prognostic models and immune therapy response and chemotherapy sensitivity

To assess the potential of high-risk and low-risk tumor samples from the model for immune therapy response, we downloaded immune phenotype scores (IPS) data for LUAD from The Cancer Immunome Atlas (TCIA, https://tcia.at/) [22, 23]. Further comparative analysis was conducted to evaluate the immune therapy potential scores of LUAD samples in different combinations of CTLA-4 and PD-1 statuses between high-risk and low-risk groups. Additionally, utilizing the online Tumor Immune Dysfunction and Exclusion (TIDE) algorithm (http://tide.dfci.harvard.edu), we assessed immune escape within the LUAD tumor microenvironment to predict tumor response to immune checkpoint inhibitors [24]. Furthermore, the validation set GSE140343 will undergo TIDE analysis to validate the predictive capabilities of the associated prognostic models in immune therapy response. Moreover, using the “oncoPredict” R package, we explored the correlation between TCGA LUAD prognostic gene scores and chemotherapy sensitivity, visualizing the results through heatmaps and boxplots. Lower-imputed drug sensitivity indicates greater sensitivity to the drug.

### Single-cell level analysis of Pre-RGs and Pro-RGs

Analysis of Pre-RGs and Pro-RGs at the single-cell level was conducted using the following steps: (1) Data preprocessing involved using “GSE131907_Lung_Cancer_raw_UMI_matrix.txt.gz” as input. The matrix was split based on “SampleName” to obtain 11 lung adenocarcinoma samples (GSM3827125 to GSM3827135). R software with the “Seurat” package was used to convert the data into Seurat objects. (2) Quality control (QC) included calculating the percentage of mitochondria and excluding low-quality cells, with parameters set as: nFeature_RNA > 500 & nCount_RNA > 1000 & nCount_RNA < 20,000 & percent.mt < 10. (3) Data normalization was performed using the “NormalizeData” function with the “LogNormalize” method and a scale factor of 10,000. (4) The top 1500 highly variable genes identified by the “FindVariableFeatures” function after QC were filtered. (5) Principal component analysis (PCA) was conducted based on these 1500 genes, followed by t-distributed stochastic neighbor embedding (tSNE) clustering for dimensionality reduction and cluster identification. (6) Significant marker genes for different clusters were identified using the “FindAllMarkers” function, setting log2|FC| to 1 and min.pct to 0.25. (7) Cell type annotations for different clusters were performed using the “SingleR” package, and the annotated results were visualized. Expression patterns of Pre-RGs and Pro-RGs were visualized, and preliminary analysis of intercellular communication in lung adenocarcinoma tissue was conducted using the “CellChat” package.

### Cohort design, sample collection, and processing

Benign lung disease and lung adenocarcinoma (LUAD) patients in the early stage of the cohort were recruited from Beijing Chest Hospital, Capital Medical University (Beijing). Informed consent was obtained from all the individuals. Benign lung disease patients were determined by surgery, image, and clinical diagnosis. Cancer patients were enrolled with confirmed pathological evidence based on the Lung Cancer Classification of Lung Tumors (WHO, 2015). Patients with multiple primary malignant neoplasms or other lung cancer types (squamous carcinoma, small cell lung cancer, etc.) were excluded from the cohort. The final cohort included 25 benign lung disease and 25 LUAD patients. This study was approved by the Ethics Committee of Beijing Chest Hospital (No. BJXK-2021-KY-12). Peripheral whole blood samples were collected from individuals before therapy or surgery using EDTA-coated vacutainer tubes. Plasma was separated within 2 h after collection by centrifuge at 1,900 g for 10 min at 4 ℃. All plasma samples were aliquoted and stored at −80 °C. CfRNAs were extracted using QIAzol Lysis Reagent (product number 79306, QIAGEN, Hilden, Germany), and DNA contamination was removed by Recombinant DNase I (RNase-free) (product number 2270 A, TAKARA, Beijing, China). The total cfRNAs libraries were prepared using our in-house protocol that improved from our previously published method, DETECTOR-seq [25]. Library quantification was performed by Qubit dsDNA HS Kit. Library fragment size and quality were checked using Agilent 2100 Bioanalyzer. Libraries were sequenced on DNBSEQ-T7 (MGI Tech.) with PE150.

### Classification model of LUAD plasma samples

We first removed cfRNA samples that did not pass quality control (7 in 50 failed), then the raw count matrix of coding genes (*n* = 19k) is normalized to TPM matrix using edgeR [26] (v3.28.1) with TMM and other default parameters in R (v3.6.0). We also removed batch effect of date and input using limma [27] (v3.42.2) package. The resulting TPM matrix was used as input of machine learning. We employed the Least Absolute Shrinkage and Selection Operator (LASSO) regression in glmnet [28] (v 4.1) package in R for classification to identify key gene features associated with LUAD. Specifically, we implemented Leave-One-Out Cross-Validation (LOOCV) to evaluate the model across different subsets of the data. The λ parameter was set as a fixed value 1*e-6 to balance the trade-off between model complexity and prediction accuracy. In the context of LUAD classification, LASSO identified a subset of coding genes that contribute most significantly to the classification between LUAD and benign lung disease groups. The AUROC and AUPR values were calculated by pROC [29] (v1.17.0.1) and PRROC [30] (v1.3.1) package in R combining all probability score of each sample.

### Statistical analysis

The statistical analyses in R (Version 4.2.2) included Spearman correlation for correlation coefficients, independent t-tests for normally distributed continuous variables, Mann–Whitney U tests for non-normally distributed variables, and ANOVA or Kruskal-Wallis tests for comparisons among multiple groups. Survival analysis employed Kaplan–Meier with log-rank tests (*P* < 0.05). Skewed numerical data were assessed using the Wilcoxon test, and categorical data were compared using chi-square or Fisher’s exact tests (*P* < 0.05).

## Results

### Chromatin accessibility analysis of LUAD ATAC-seq data

**Fig. 1:**
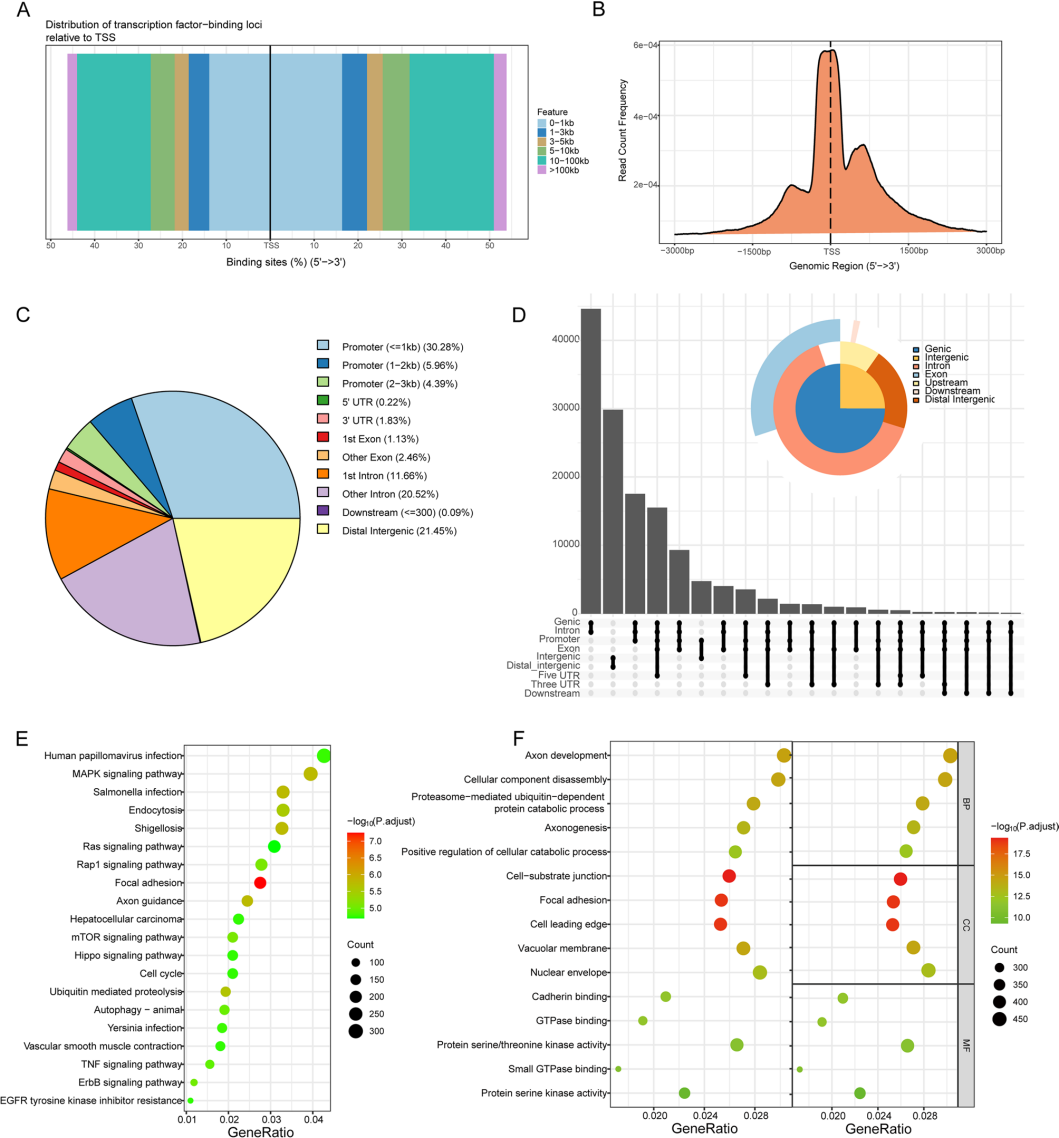
Chromatin Accessibility and Genomic Characterization (**A**) Distribution of ATAC-seq Peaks Relative to TSS; (**B**) Enrichment of Peaks in the TSS region; (**C**)Location distribution of Peaks on the genome; (**D**)Relative proportions of gene coding regions, intergenic regions, introns, exons, and upstream and downstream regions; (**E**) KEGG Pathway Enrichment Analysis of Genes Associated with ATAC-seq TSS Binding Sites; (**F**) GO Functional Enrichment Analysis of Genes Associated with ATAC-seq TSS Binding Sites

ATAC-seq identified chromatin accessible regions by mapping the genomic coordinates of detected peaks across 24 human chromosomes, including X and Y chromosomes (File S1, Figure S2A). The distribution of these peaks was relatively uniform across 23 chromosomes, except for the Y chromosome. Most regions on each chromosome exhibited peak coverage, with relatively lower coverage observed on the short arms of chromosomes 13, 14, 15, and 22. Figure 1A shows that peaks predominantly clustered within 0–1 kb and 10–100 kb from TSS. Furthermore, results from Fig. 1B and Supplementary Figure S2B indicate that the majority of open chromatin regions’ peaks were closely adjacent to TSS, consistent with chromatin accessibility characteristics. This affirms the reliability and authenticity of the sequencing data, suggesting interactions between TSS and transcription factors.

### Genomic characterization and enrichment analysis of ATAC peaks

Using annotation files, we annotated genomic coordinates corresponding to the identified peaks. Figure 1C illustrates the distribution across different genomic components, with the highest proportion observed in promoter regions: 30.28% within 1 kb around TSS, 5.96% between 1 and 2 kb, and 4.39% between 2 and 3 kb. Intergenic regions accounted for 21.45%, followed by other intronic peaks at 20.52%. This distribution corresponds to two major types of open chromatin regions: promoter regions upstream of genes and distal regulatory elements (enhancers and silencers). Figure 1D summarizes the relative enrichment proportions of coding regions, intergenic regions, introns, exons, and upstream/downstream regions, suggesting diverse positional features of peaks in open chromatin regions. This indirectly elucidates the diversity and complexity of biological regulation.

Figure 1E and F depict KEGG pathway enrichment analysis and GO functional enrichment results for genes corresponding to ATAC-seq TSS binding sites. KEGG analysis revealed significant enrichment in several cancer-related signaling pathways, including the MAPK signaling pathway, Rap1 signaling pathway, Ras signaling pathway, and ErbB signaling pathway. Additionally, GO enrichment results showed that these genes are predominantly involved in biological processes (BP) such as Wnt signaling pathway, Wnt-mediated intercellular signaling, cellular component disassembly, and axon development. Furthermore, they are associated with metabolic activities such as actin binding, phospholipid binding, and molecular adaptor activity. Overall, the enrichment results indicate that most TSS binding sites are located within genes involved in cellular morphogenesis, intracellular transport, kinase activity, and regulation of functions and metabolic pathways related to infection, cancer, and cell cycle. These functional pathways and metabolic processes have been implicated in cancer development.

### Differential expression analysis of TCGA and GTEx RNA-seq data

**Fig. 2:**
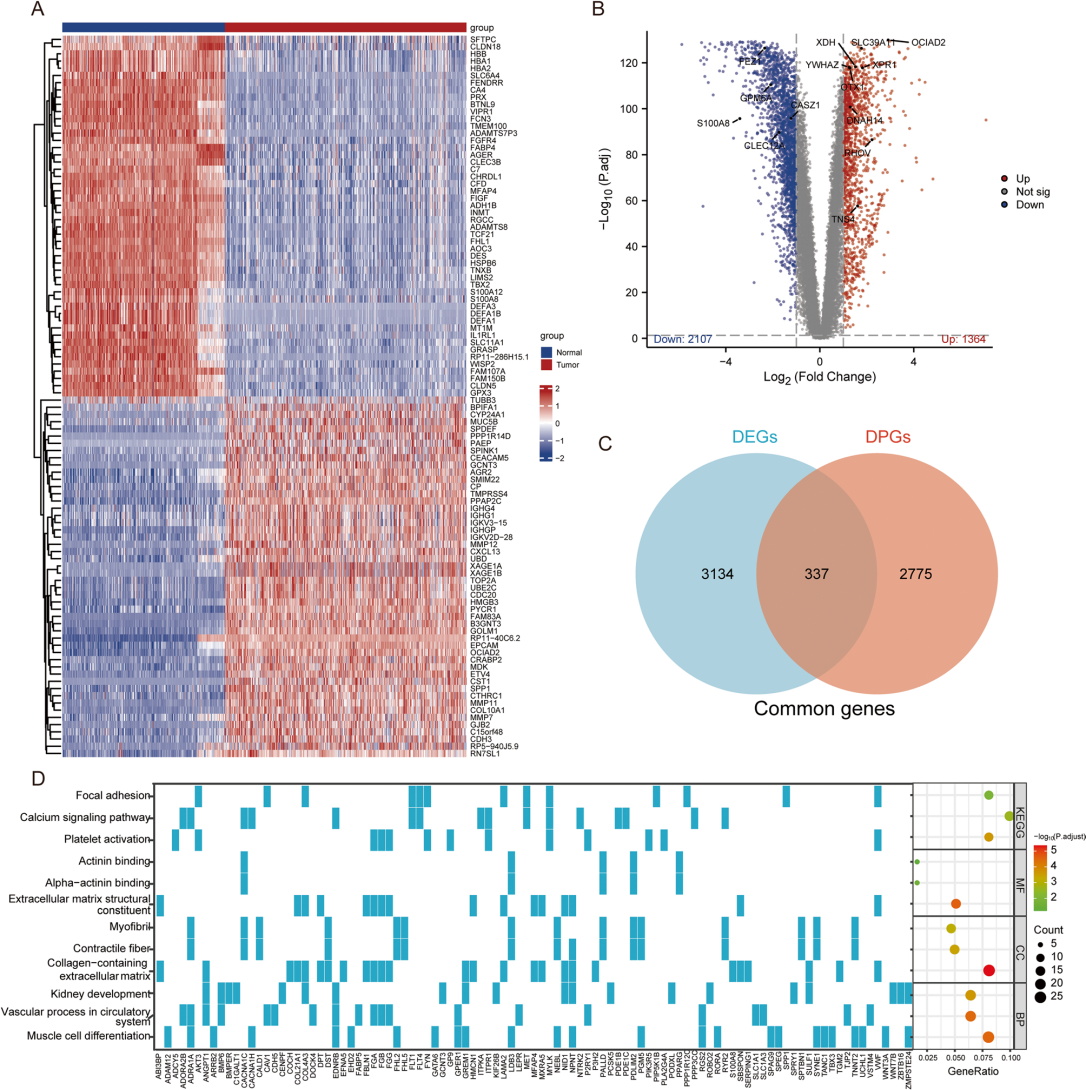
Differential Expression and Peak Analysis in LUAD (**A**) Heatmap Visualization of Top 100 DEGs in LUAD (Red: high expression); (**B**) Volcano Plot of DEGs between LUAD Tumor and Normal Samples; (**C**) Intersection of DPGs and DEGs; (**D**) Functional Enrichment Analysis of CGs from DPGs and DEGs in LUAD

RNA-seq was conducted on a total of 515 LUAD tumor samples and 347 normal samples obtained from the TCGA and GTEx databases to analyze differential gene expression. Through this analysis, a total of 3471 DEGs were identified (Supplementary material 2, Table S1). The top 100 significantly differentially expressed genes (50 upregulated and 50 downregulated) were selected for visualization as a heatmap, as shown in Fig. 2A. To further investigate the significance of these differentially expressed genes, a volcano plot was generated to depict the filtered set of upregulated genes (*N* = 1364) and downregulated genes (*N* = 2107), as illustrated in Fig. 2B.

### Analysis of differential ATAC-seq peaks between stage I and stage II-IV LUAD

Through differential analysis, a total of 4612 differential peaks between TNM stage I and stage II-IV were identified, comprising 3563 upregulated and 1049 downregulated peaks (Supplementary material 2, Table S2). Annotation of these differential peaks revealed 3113 genes associated with DPGs. Intersection of DPGs with DEGs yielded 337 CGs (Fig. 2C, Supplementary material 2, Table S3). Subsequently, GO functional enrichment analysis and KEGG pathway enrichment analysis were performed on these CGs (Fig. 2D, Supplementary material 2, Table S4). These genes are implicated in various biological processes and signaling pathways, including muscle cell differentiation, kidney development, vascular processes in the circulatory system, platelet activation, focal adhesion, and calcium signaling pathway. Additionally, metabolic functions such as alpha-actinin binding, actinin binding, extracellular matrix structural constituents, and constituents were identified. Overall, these findings suggest that CGs may play crucial roles in the occurrence and progression of LUAD.

### Random forest and LASSO analysis for Pre-RGs

**Fig. 3:**
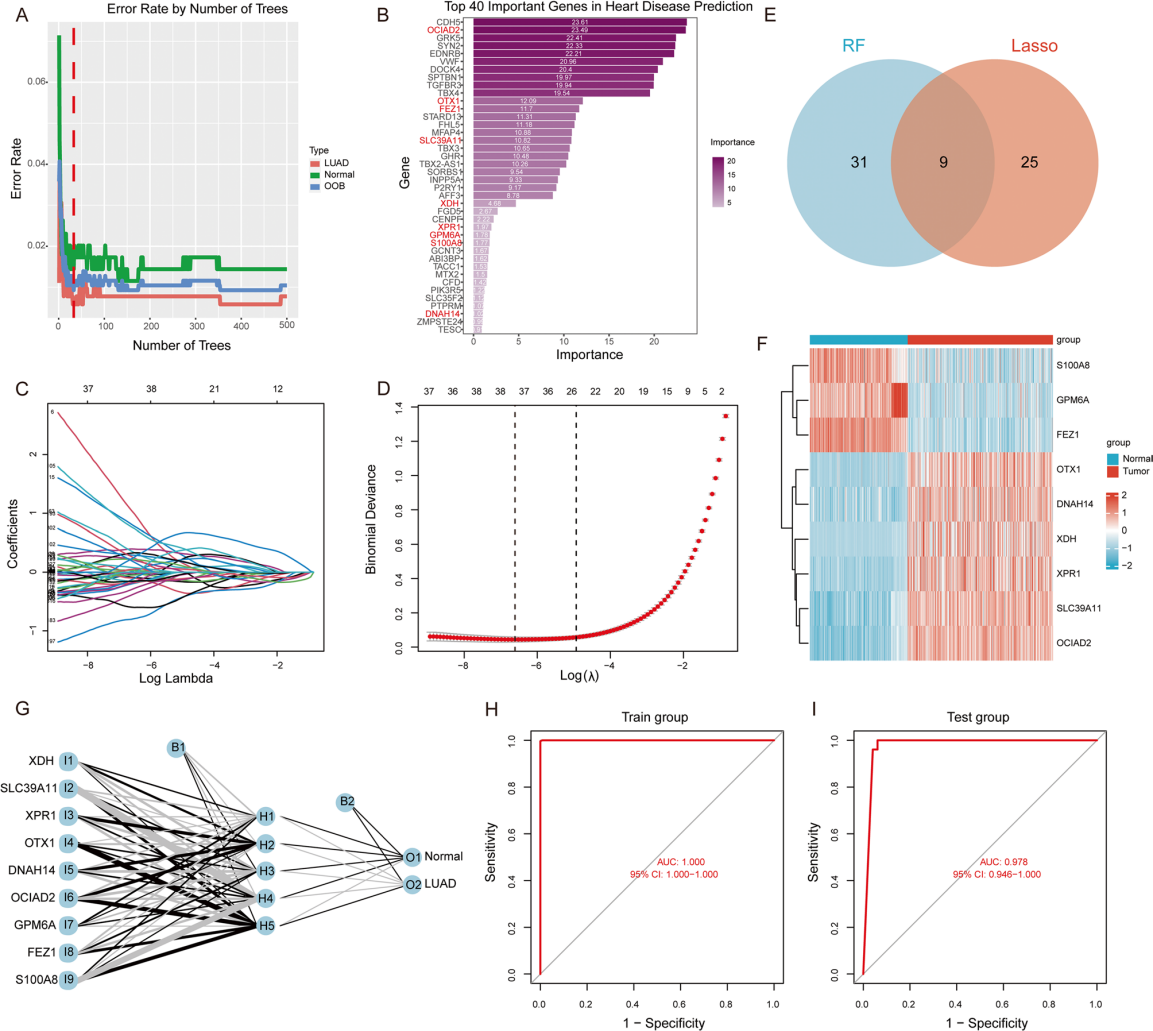
Identification and Validation of Pre-RGs for LUAD Diagnostic Model (**A**) Cross-Validation Error Minimization in Random Forest Model with 33 Trees; (**B**) Importance Ranking of 40 Representative Genes from Random Forest Model; (**C**) LASSO Model Selection of Penalty Parameter (λ) for Optimal Gene Screening; (**D**) LASSO Model Path for Selection of 34 Genes; (**E**) Intersection of Genes Identified by Random Forest and LASSO Models; (**F**) Expression Comparison of 9 Intersecting Genes in LUAD and Normal Tissues; (**G**) Structure of ANN model for LUAD Diagnostic Predictive Model; Diagnostic Performance of LUAD Predictive Model in Training Cohort (**H**) and Validation Cohort (**I**)

The 337 CGs were inputted into a random forest model, which minimized the cross-validation error across 33 trees, and the results were visualized (Fig. 3A). Subsequently, 40 representative genes were identified based on the importance ranking from the random forest model and visualized accordingly (Fig. 3B). Additionally, the LASSO model was applied to screen the 337 CGs, with the penalty parameter (λ) set to its minimum value of 0.001348504 (Fig. 3C, D). The LASSO model ultimately identified 34 genes (Supplementary material 2, Table S5). Furthermore, a set of 9 genes were identified as intersecting between the random forest and LASSO models (*S100A8*, *GPM6A*, *FEZ1*, *OTX1*, *DNAH14*, *XDH*, *XPR1*, *SLC39A11*, *OCIAD2*) for establishing Pre-RGs in subsequent predictive modeling (Fig. 3E). Comparisons with normal tissues showed significant upregulation of *OTX1*, *DNAH14*, *XDH*, *XPR1*, *SLC39A11*, and *OCIAD2* in LUAD tissues, while *S100A8*, *GPM6A*, and *FEZ1* were significantly downregulated in LUAD tissues (Fig. 3F).

### Building and validation of LUAD Pre-RGs predictive model

Using a neural network algorithm, a diagnostic predictive model for 9 Pre-RGs genes was successfully constructed. The model consists of input, hidden, and output layers (Fig. 3G). The model formula is defined as follows: Neural Operating System = ∑ (Gene Score × Gene Weight) (Gene weights detailed in the Supplementary material 2, Table S6). The model demonstrated excellent diagnostic performance for LUAD in both training and validation cohorts, with an AUC of 1.000 (95% CI: 1.000–1.000) in the training set (Fig. 3H), indicating perfect predictive accuracy. Additionally, when applied to the validation set, the model achieved an AUC of 0.978 (95% CI: 0.946-1.000) (Fig. 3I), confirming the high reliability and stability of the LUAD 9 Pre-RGs diagnostic model on independent validation data (Tables 1 and 2).

**Table 1:** The accuracy of the model in the training group

Model prediction TCGA pathology	Normal	LUAD	Total
Normal	346	1	347
LUAD	1	514	515

**Table 2:** The accuracy of this model in the validation group

Model prediction GEO pathology	Normal	LUAD	Total
Normal	44	5	49
LUAD	0	51	51

### Construction of LUAD prognostic model

**Fig. 4:**
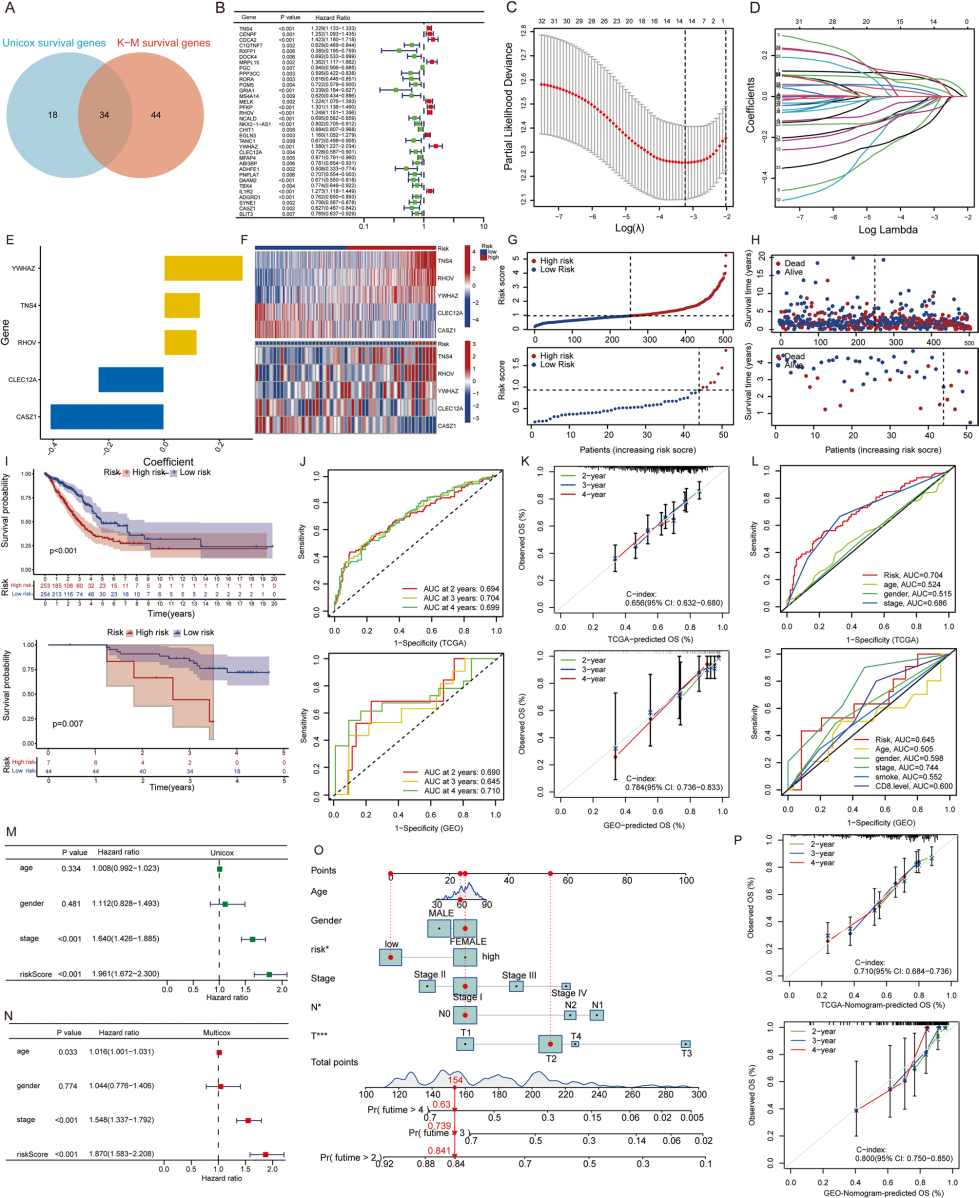
Construction and Validation of LUAD Prognostic Model (**A**) Intersection of Univariate Cox and Kaplan-Meier Survival Analysis of CGs, visualized using a forest plot of intersecting genes (**B**); (**C**-**D**) LASSO Model Selection and Path for Dimensionality Reduction; (**E**) Bidirectional Stepwise Regression Identification of 5 Final Pro-RGs. (**F**) Heatmap of Differential Expression of Prognostic Genes Between High and Low-Risk Groups (Red: high expression); Risk Score Plot (**G**) and Survival Status Plot (**H**) Showing Mortality Rates and Survival Times in High and Low-Risk Groups. (top: TCGA data; bottom: GEO data); (**I**) Kaplan-Meier Survival Curves for High and Low-Risk Groups Across Cohorts (top: TCGA data; bottom: GEO data); AUC Values (**J**) and Calibration Curves (**K**) for Predicting 2, 3, and 4-Year Overall Survival in Training and Validation Sets (top: TCGA data; bottom: GEO data); (**L**) Comparison of Predictive Efficacy for Overall Survival Among Different Clinical Indicators(top: TCGA data; bottom: GEO data); Univariate (**M**) and multivariate (**N**) cox Analysis of Model Risk Scores. (**O**) Nomogram for predicting the 2-, 3-, and 4-year OS of LUAD patients. (**P**) Calibration Curves for Model Predictions Using TCGA and GSE140343 Data

We conducted univariate cox analysis (*P* < 0.01) and Kaplan-Meier analysis (*P* < 0.05) on 337 CGs, selecting 34 genes through screening (Fig. 4A, B). Subsequently, we performed dimensionality reduction using LASSO (Fig. 4C, D), resulting in 14 genes. Through bidirectional stepwise regression, we identified 5 final prognostic-related genes (Tensin 4 (*TNS4*), Ras Homolog Family Member V (*RHOV*), Tyrosine 3-Monooxygenase/Tryptophan 5-Monooxygenase Activation Protein Zeta (*YWHAZ*), C-Type Lectin Domain Family 12 Member A (*CLEC12A*), Castor Zinc Finger 1 (*CASZ1*)) (Fig. 4E). Concurrently, we developed a multivariable Cox proportional hazards model to calculate risk scores for each sample, defined by the formula: Risk score = (0.131070 * *TNS4*) + (0.119435 * *RHOV*) + (0.285820 * *YWHAZ*) - (0.238335 * *CLEC12A*) - (0.411529 * *CASZ1*). Using this formula, we computed risk scores for training and validation cohorts. Subsequently, based on median scores in the training cohort, participants were stratified into high and low-risk groups. A heatmap depicted differential expression of *TNS4*, *RHOV*, *YWHAZ*, *CLEC12A*, and *CASZ1* between high and low-risk groups in the TCGA training and GEO validation cohorts (Fig. 4F). Furthermore, risk score plots indicated higher mortality rates and shorter survival times in patients with higher scores (Fig. 4G, H). Across training, testing, and combined patient cohorts, patients in the high-risk group exhibited poorer overall survival (OS) compared to the low-risk group (*P* < 0.01) (Fig. 4I). Due to missing 1-year and 5-year prognostic data in the validation set, OS was predicted for 2, 3, and 4 years in the training set. The model achieved AUC values of 0.694, 0.704, and 0.699 for predicting 2-year, 3-year, and 4-year OS in TCGA LUAD patients, respectively (Fig. 4J). This underscores the prognostic value of the model for LUAD OS. Moreover, in the validation set, AUC values were 0.690, 0.645, and 0.710, demonstrating robust predictive performance. Calibration curves for 2-year, 3-year, and 4-year survival probabilities in both training and validation sets showed satisfactory agreement between predicted and observed values (Fig. 4K). The C-index was 0.656 (95% CI: 0.632–0.680) in the training set and 0.784 (95% CI: 0.736–0.833) in the validation set, affirming the model’s ability to predict OS based on different risk scores in LUAD. Compared with other clinical indicators, risk score showed superior predictive efficacy for OS outcomes relative to age, gender, and staging factors in the training set, while in the validation set, its predictive power was stronger compared to age, gender, smoking, and CD8 level, but weaker than staging factors (Fig. 4L).

Furthermore, based on TCGA data, single-factor and multivariate Cox analyses of model risk scores indicated that the risk score was an independent prognostic factor for LUAD OS (Fig. 4M, N). In the validation set, the risk score demonstrated significance as a single factor, while multivariate Cox analysis showed no difference, possibly due to collinearity between risk score and other factors such as stage. Removal of stage as a factor revealed closer significant differences in prognostic scores (File S1, Figure S3A). Additionally, with only 51 cases in the GEO dataset, combining TCGA and GEO data for further single-factor and multi-factor analyses confirmed the model’s risk score as an independent prognostic factor for OS (Figure S3B-F). Overall, integrating clinical features of entire TCGA LUAD patient cohort, including prognostic model risk scores, T, N (excluding M due to M0 status), age, gender, and stage, into a Cox proportional hazards model enabled construction of a column chart (Fig. 4O). Calibration curves using TCGA and GSE140343 data for 2, 3, and 4-year model predictions demonstrated satisfactory consistency between predicted and observed values (Fig. 4P).

### Correlation of LUAD Pro-RGs model risk score with immune infiltration and cancer stem cell index

**Fig. 5:**
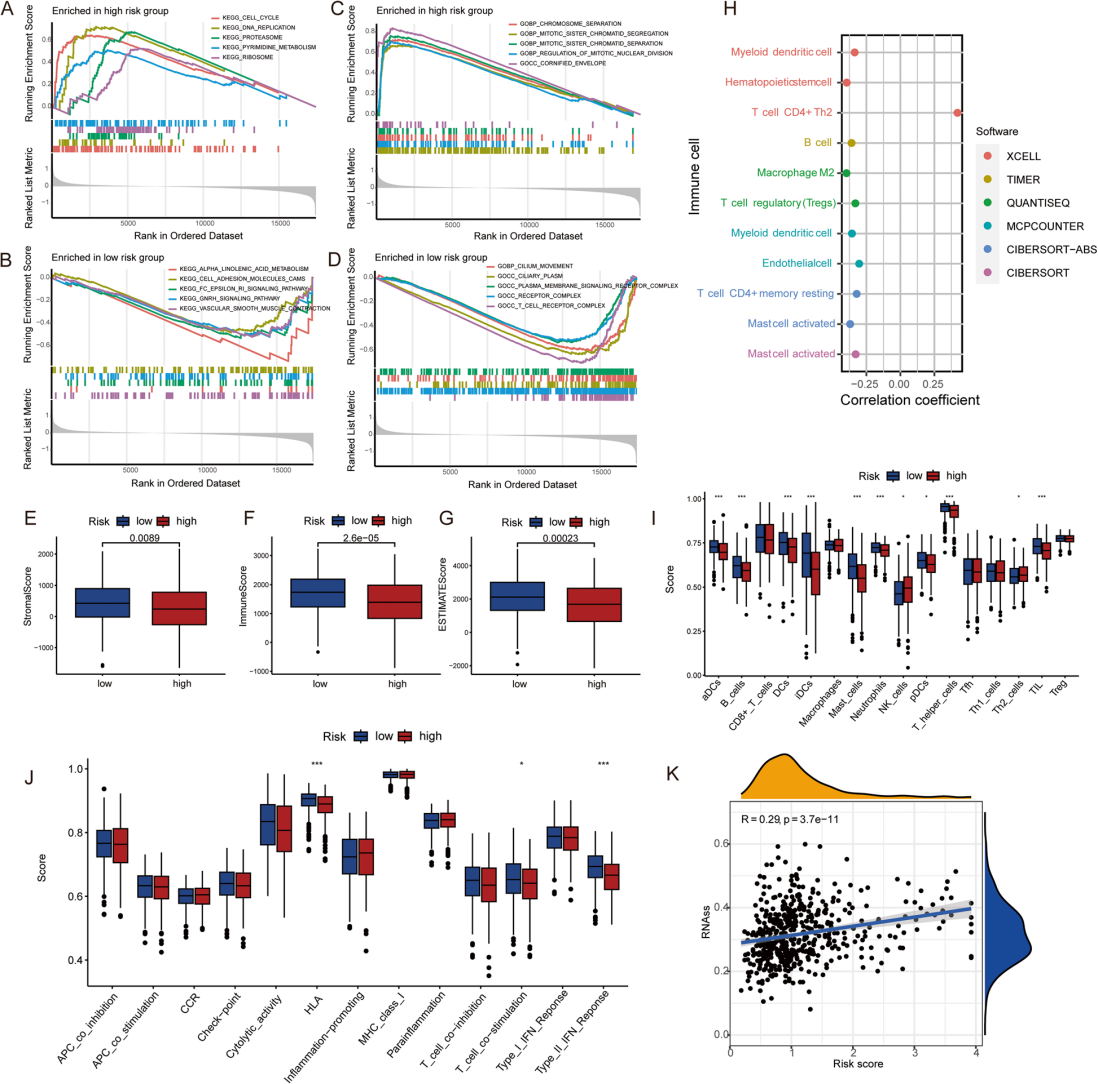
Correlation of LUAD Pro-RGs Model risk Score with Immune Infiltration and Cancer Stem Cell Index GSEA Enrichment Analysis of High-Risk (**A**, **B**) and Low-Risk (**C**, **D**) Score Group (KEGG Pathways and GO Terms); Comparison of Stromal Scores (**E**), Immune Scores (**F**) and ESTIMATE Scores (**G**) Between High and Low-Risk Groups; (**H**) Correlation Between Immune Cell Infiltration and Risk Scores; (**I**) ssGSEA Analysis of Immune Cell Infiltration in High and Low-Risk Groups; (**J**) Immune Function Analysis Between High and Low-Risk Groups; (**K**) Correlation Between Prognostic Model Risk Score and Cancer Stem Cell Index

Using GSEA, functional enrichment analysis was performed on two groups with high and low prognostic gene scores. The high-risk score group was primarily enriched in cell cycle, DNA replication, proteasome, pyrimidine metabolism, ribosome KEGG pathways, and chromosome separation, mitotic sister chromatid segregation, mitotic sister chromatid separation, regulation of mitotic nuclear division GO terms (Fig. 5A, B). Conversely, the low-risk group was enriched in alpha-linolenic acid metabolism, cell adhesion molecules (CAMs), FC epsilon RI signaling pathway, GnRH signaling pathway, and vascular smooth muscle contraction KEGG pathways, as well as cilium movement, ciliary plasm, and plasma membrane signaling receptor complex GO terms (Fig. 5C, D; Supplementary material 2, Table S7, S8).

Compared to the low-risk group, the high-risk group exhibited significantly lower TME scores (including stromal score, immune score, and ESTIMATE score) (Fig. 5E, F, G). A lower immune score indicates reduced immune cell infiltration, typically associated with a poorer immune response and worse prognosis, potentially suggesting a reduced response to immunotherapy, which may partly explain the lower overall survival rate in the high-risk group. Various software tools assessed the correlation between immune infiltration and the risk scores of the prognostic model, with results showing correlations ≥ 0.3 (Fig. 5H, details in File S1, Figure S4). Among these, only T cell CD4 + Th2 showed a positive correlation with the risk score, while Mast cell activated, T cell CD4 + memory resting, Endothelial cell, Myeloid dendritic cell, T cell regulatory (Tregs), Macrophage M2, B cell, and Hematopoietic stem cell showed a negative correlation with the prognostic gene risk score. ssGSEA analysis revealed significant differences in aDCs, B cells, DCs, iDCs, Mast cells, pDCs, T helper cells, and TIL between high- and low-risk groups, with higher infiltration in the low-risk group. Additionally, NK cells and Th2 cells showed differences, with higher infiltration in the high-risk group (Fig. 5I, Supplementary material 2, Table S9).

Immune function analysis indicated differences in HLA, T cell co-stimulation, and Type II IFN response between high and low-risk scores, with stronger immune-related functions in the low-risk group (Fig. 5J). Cancer stem cells (CSCs), characterized by self-renewal, multipotency, and tumor-initiating properties, play critical roles in tumorigenesis, progression, recurrence, and drug resistance. The correlation between the prognostic model risk score and the CSC index was found to be moderately positive and significant (*R* = 0.29, *P* = 3.7e − 11) (Fig. 5K).

### Correlation of LUAD prognostic gene scores with tumor mutation burden and immune checkpoint genes

**Fig. 6:**
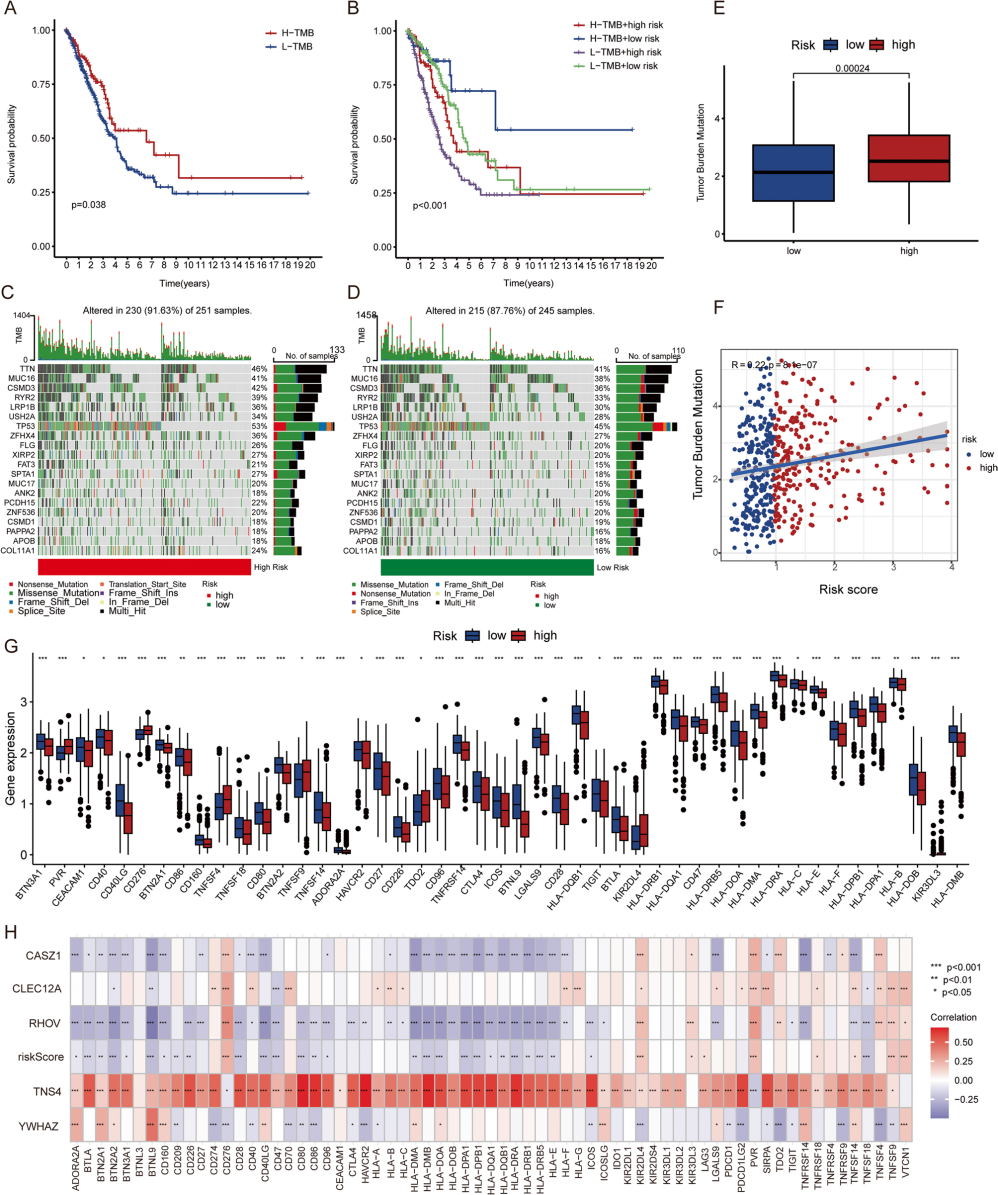
Correlation of LUAD Prognostic Gene Scores with Tumor Mutation Burden and Immune Checkpoint Genes (**A**) Prognostic Analysis of High TMB and Low TMB Groups; (**B**) Stratification by TMB and Prognostic Model Risk Scores; (**C**) Mutation Incidence in High and Low-Risk Groups; Differential Analysis of TMB Scores Between High (**D**) and Low-Risk (**E**) Groups; (**F**) Correlation Between Prognostic Model Risk Score and TMB Score; (**G**) Expression Levels of Immune Checkpoint Genes in High and Low-Risk Groups; (**H**) Correlation Analysis Between Pro-RGs, Risk Score, and Immune Checkpoint Genes

The overall tumor mutation profile of TCGA LUAD patients revealed that the predominant variant classification was Missense Mutation, with most variant types being SNPs. The top 10 mutated genes were *TTN*, *MUC16*, *CSMD3*, *RYR2*, *LRP1B*, *TP53*, *USH2A*, *ZFHX4*, *FLG*, and *KRAS* (File S1, Figure S5A-F). Prognostic analysis of high TMB and low TMB groups showed that the high TMB group had better outcomes (Fig. 6A). Stratification by TMB and prognostic model risk scores indicated that patients with both high TMB and low-risk scores had the best prognosis (Fig. 6B), suggesting that the prognostic model risk score might have a greater impact on prognosis.

Describing the mutation burden in high and low-risk groups according to the prognostic model risk score, the overall mutation incidence was slightly higher in the high-risk group (91.63%) compared to the low-risk group (87.76%). Notably, the *TP53* mutation rate was higher in the high-risk group (53%) than in the low-risk group (45%) (Fig. 6C, D). Differential analysis showed that the TMB score was significantly elevated in the high-risk group (*P* = 0.00024) (Fig. 6E), and there was a positive correlation between the prognostic model risk score and TMB score (*R* = 0.22, *P* = 8.1e − 07) (Fig. 6F).

The expression levels of immune checkpoint genes (ICGs) are closely related to the clinical efficacy of immune checkpoint blockade therapy [31, 32]. Further analysis of the relationship between prognostic gene scores and ICGs revealed that, except for a few genes (*PVR*, *TNFSF9*, *TDO2*, *KIR2DL4*) that were higher in the high-risk group, most ICGs were more highly expressed in the low-risk group (Fig. 6G). This suggests that LUAD patients with low-risk scores may respond better to immunotherapy.

Correlation analysis between ICGs and the five genes in the prognostic model, as well as the model risk score, showed that *CASZ1*, *CLEC12A*, *RHOV*, and the riskScore were negatively correlated with most ICGs, while *TNS4* was positively correlated with most ICGs (Fig. 6H, correlation results in the Supplementary material 2, Table S10).

### Correlation of LUAD prognostic model with immunotherapy response and chemotherapy sensitivity

**Fig. 7:**
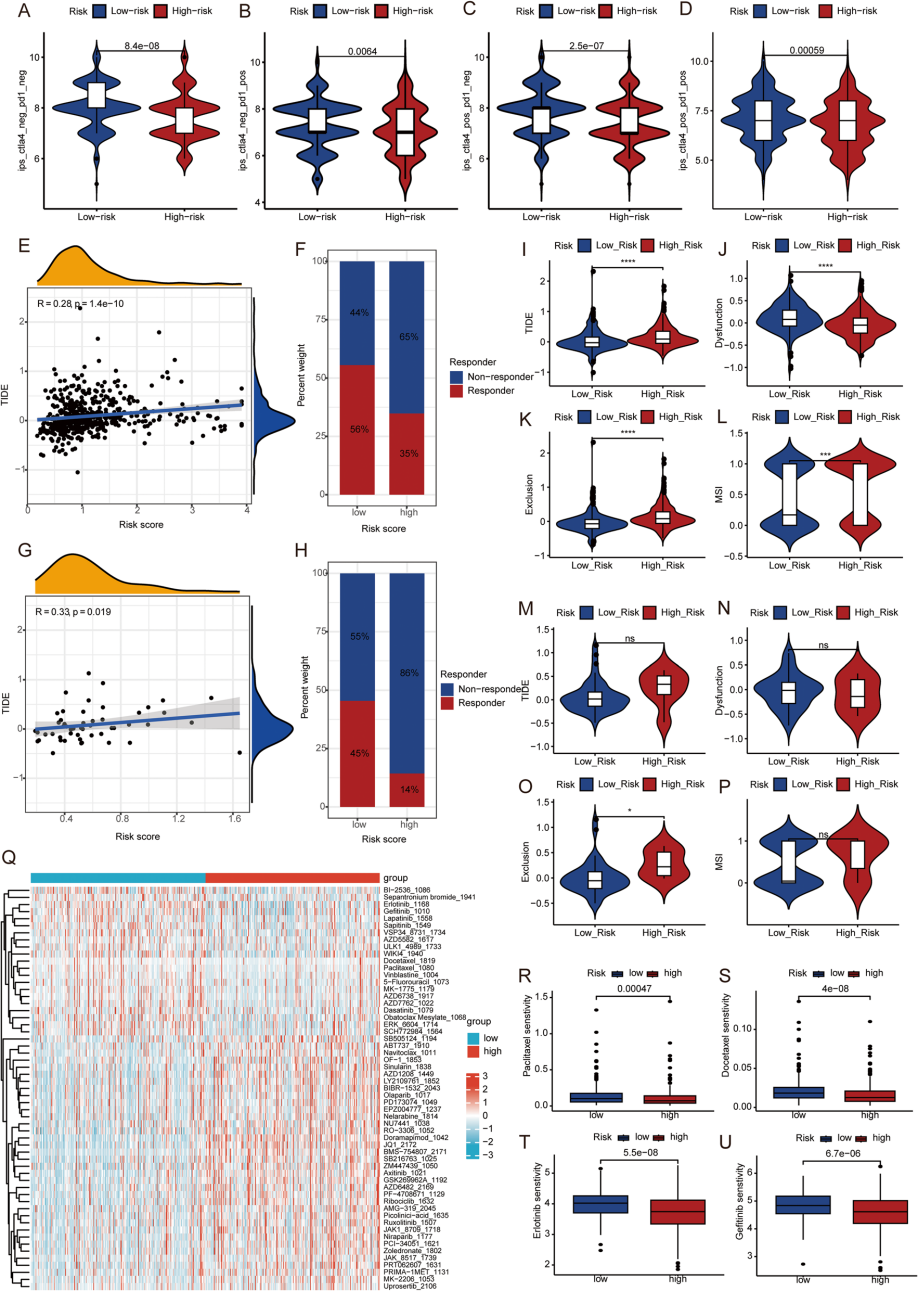
Correlation of LUAD Prognostic Model with Immunotherapy Response and Chemotherapy Sensitivity (**A**-**D**): Immunotherapy Response Prediction Based on TICA analysis; (**E**-**H**): TIDE Score Analysis and Response Distribution (**E**-**F** for TCGA data, **G**-**H** for GEO data); (**I**-**P**) Differential Analysis of Immune Scores Between High and Low-Risk Groups (**I**-**L** for TCGA data, **M**-**P** for GEO data); (**Q**-**U**) Chemotherapy Sensitivity Analysis

Based on TICA data, analysis of immunotherapy responses revealed that the average Immunophenoscore (IPS) values were significantly higher in the low-risk group compared to the high-risk group across all four immunotherapy scenarios. Specifically, in the IPS_ CTLA-4_neg_ PD-1_neg scenario, which represents dual resistance to anti-CTLA-4 and anti-PD-1 therapies, lower IPS scores are associated with a higher likelihood of treatment failure. Consistently, patients in the high-risk group exhibited lower IPS scores, suggesting a greater propensity for therapeutic resistance (Fig. 7A). Furthermore, a significantly higher predicted positive immune response was observed in the low-risk group for anti-PD-1 therapy (Fig. 7B), anti-CTLA-4 therapy (Fig. 7C), and the combination of both therapies (Fig. 7D) (*P* < 0.001). These results indicate that immunotherapy is more effective in patients with low prognostic risk scores.

Further analysis using TCGA LUAD TIDE data demonstrated a positive correlation between the model risk score and TIDE score (*R* = 0.28, *P* = 1.4e − 10) (Fig. 7E). The TIDE algorithm divided patients into non-responders and responders, revealing that the proportion of responders was higher in the low-risk group (56%) compared to the high-risk group (35%) (Fig. 7F). This consistent finding suggests greater sensitivity to immunotherapy in the low-risk group.

Similarly, validation with the GSE140343 dataset showed a positive correlation between the model risk score and TIDE score (*R* = 0.33, *P* = 0.019) (Fig. 7G), with a higher proportion of responders in the low-risk group (45%) compared to the high-risk group (14%) (Fig. 7H), corroborating the TCGA findings.

Differential analysis between high and low-risk groups revealed significant differences in TIDE, Exclusion, and MSI scores, with higher means in the high-risk group, while Dysfunction had a lower mean in the high-risk group (Fig. 7I-P). Additionally, significant differences were observed for IFNG, Merck18, CD274, MDSC, CAF, and TAM M2 between the groups. IFNG, Merck18 and TAM M2 had higher mean values in the low-risk group, whereas CD274, MDSC, and CAF had higher means in the high-risk group (File S1, Figure S6A-F), indicating greater immunotherapy sensitivity in the low-risk group. In the validation set, Exclusion and CAF showed higher mean values in the high-risk group, while TAM M2 had higher mean values in the low-risk group, consistent with the training set. No differences were observed for the other indicators (Figure S6G-L). Interestingly, although TAM M2 cells are generally associated with immunosuppression and poor prognosis, higher TAM M2 levels were observed in the low-risk group in our study. This paradoxical finding may be attributed to the high heterogeneity and plasticity of the tumor microenvironment. In certain contexts, M2-like macrophages can be reprogrammed to support antitumor immunity or participate in tissue remodeling rather than promoting immune evasion. Additionally, variations in estimation algorithms and the potential interactions with other immune components might also contribute to this observation. We also analyzed drug sensitivity to common LUAD targeted and chemotherapeutic agents across different risk score groups (Fig. 7Q). Paclitaxel (Fig. 7R), Docetaxel (Fig. 7S), Erlotinib (Fig. 7T), and Gefitinib (Fig. 7U) showed higher sensitivity in the low-risk group (other drugs detailed in Supplementary material 2, Table S11).

#### Single-Cell analysis of Pre-RGs and Pro-RGs

**Fig. 8:**
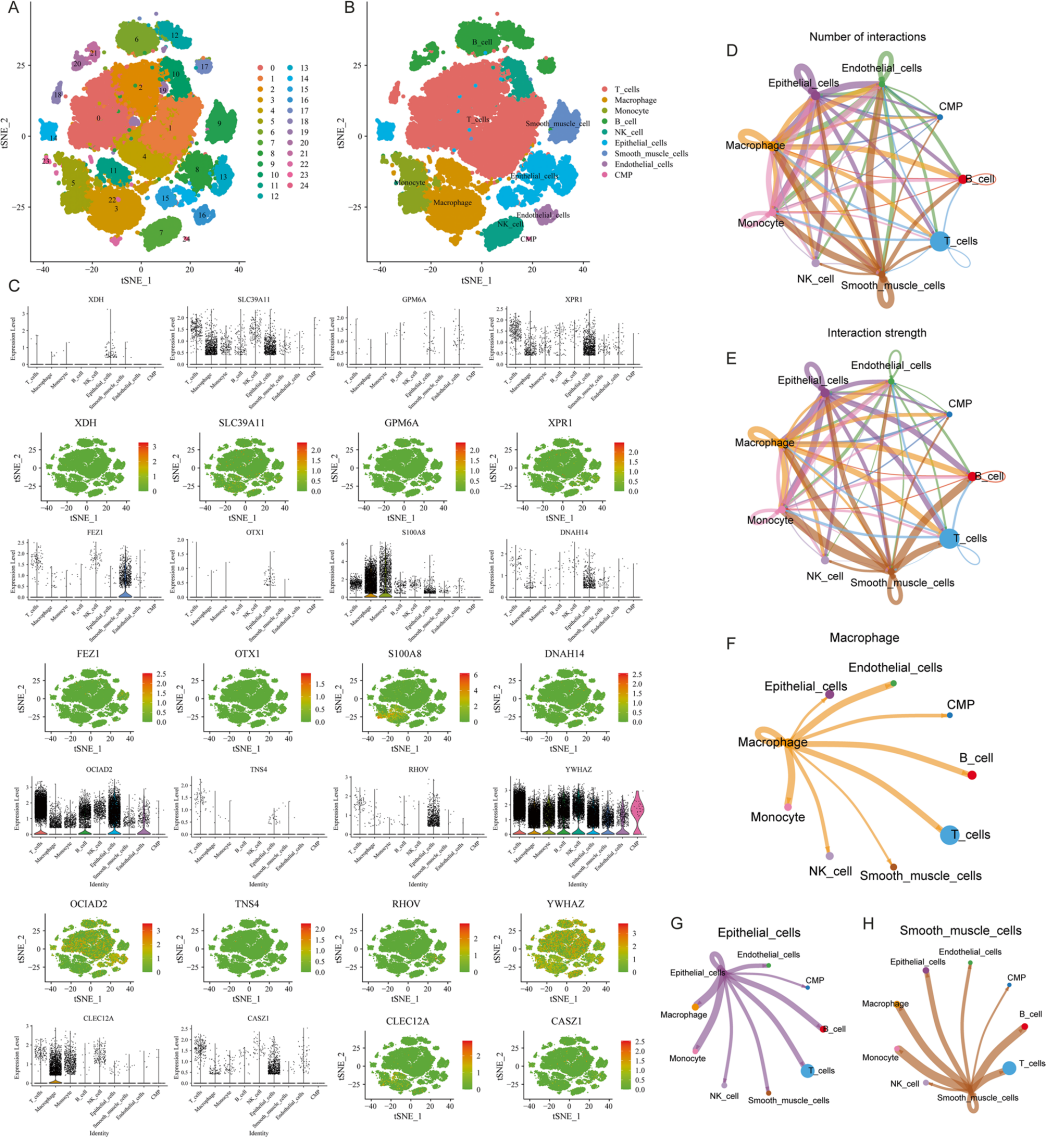
Single-Cell Analysis of Gene Expression and Cell Communication in LUAD Tumor Microenvironment Identification of 24 Subpopulations through Clustering Analysis (**A**) and Annotation of Cell Subtypes (**B**); Gene Expression Patterns across Different Cell Types (**C**) and Analysis of Cell Interaction Strength (cellNetworkCount) (**D**) and Quantity (cellNetworkWeight) (**E**); (**F**-**H**) Role of Macrophages, Epithelial Cells, and Smooth Muscle Cells in Cell Communication

Correlation analysis between 14 genes and immune cells (File S1, Figure S7) revealed that these genes are associated with various immune cells. Thus, we further examined the expression of these 14 genes at the single-cell level. After quality control, data filtering, dimensionality reduction, and clustering, 24 subpopulations were identified (Fig. 8A, File S1, Figure S8A-I). These were annotated into 9 cell subtypes: T cells, Macrophages, Monocytes, B cells, NK cells, Epithelial cells, Smooth muscle cells, Endothelial cells, and CMP (Fig. 8B).

The bar chart (Fig. 8C) shows that genes *XDH*, *GPM6A*, *OTX1*, and *TNS4* have low expression, primarily in Epithelial cells. Genes *XPR1*, *DNAH14*, *RHOV*, and *CASZ1* have moderate expression, also mainly in Epithelial cells. The *FEZ1* gene is predominantly expressed in Smooth muscle cells, while *SLC39A1* is primarily expressed in Macrophages and Epithelial cells. *S100A8* is concentrated in T cells, Macrophages, and Monocytes, with minor expression in Epithelial cells. Similarly, *CLEC12A* is mainly expressed in Macrophages and Monocytes, with low expression in other cells. *OCIAD2* and *YWHAZ* are expressed in all cell types except CMP, with *YWHAZ* having higher overall expression.

Based on these data, we also conducted preliminary cell communication studies to examine the interactions of annotated cell subtypes within the tumor microenvironment. Analysis of cellNetworkCount (Fig. 8D) and cellNetworkWeight (Fig. 8E) indicated that Macrophages, Epithelial cells, and Smooth muscle cells have relatively high interaction strength and quantity with other cells in the microenvironment, suggesting that these three cell types play crucial roles in the tumor microenvironment (Fig. 8F-H).

### Expression differences and functional analysis of Pre-RGs and Pro-RGs

**Fig. 9:**
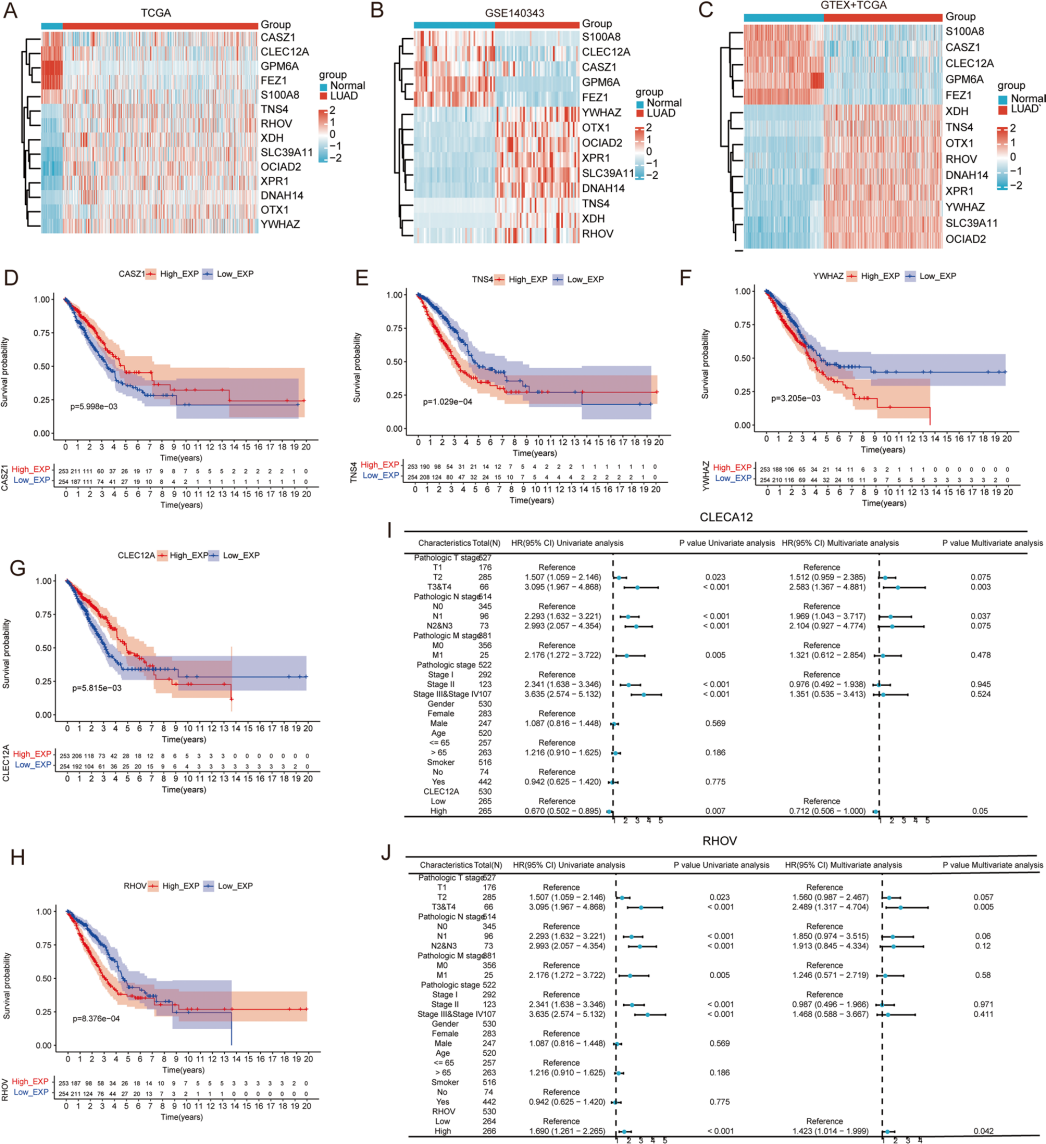
Expression and Prognostic Analysis of Pre-RGs and Pro-RGs in LUAD (**A**-**C**) Expression heatmaps of 14 Pre-RGs and Pro-RGs across TCGA, GEO, and GTEx + TCGA datasets (Red: high expression). (**D**-**H**) Kaplan-Meier plots showing OS based on expression levels of Pro-RGs in LUAD (**I**, **J**) Independent prognostic analysis of CLEC12A and RHOV in TCGA LUAD patients

We used heatmaps to illustrate the expression of 14 Pre-RGs and Pro-RGs across TCGA, GEO, and GTEX + TCGA datasets, revealing consistent results. Compared to normal or adjacent non-tumor tissues, *S100A8*, *CLEC12A*, *CASZ1*, *GPM6A*, and *FEZ1* were downregulated in tumor tissues, while *YWHAZ*, *OTX1*, *OCIAD2*, *XPR1*, *SLC39A11*, *DNAH14*, *TNS4*, *XDH*, and *RHOV* were upregulated (Fig. 9A-C). Additionally, we plotted K-M plots (Fig. 9D-H) and conducted independent prognostic analysis for the five Pro-RGs (*TNS4*, *RHOV*, *YWHAZ*, *CLEC12A*, and *CASZ1*) based on TCGA data, revealing that only *CLEC12A* and *RHOV* were independent prognostic factors for LUAD OS (Fig. 9I, J). *TNS4*, *YWHAZ*, and *CASZ1* were not significant (File S1, Figure S9A-C).

The Lung Cancer Explorer (LCE) (https://lce.biohpc.swmed.edu/) is a powerful platform for analyzing gene expression and clinical features in lung cancer [33]. Using the LCE database, we performed a meta-analysis of mRNA expression differences and prognosis for Pre-RGs and Pro-RGs in LUAD and normal lung tissues, displaying the results via forest plots (File S1, Figure S10A-N). The results demonstrate that *S100A8*, *CLEC12A*, *CASZ1*, *GPM6A*, and *FEZ1* are significantly downregulated, while *YWHAZ*, *OTX1*, *OCIAD2*, *XPR1*, *SLC39A11*, *DNAH14*, *TNS4*, *XDH*, and *RHOV* are upregulated in tumor tissues, aligning with prior findings. Additionally, meta-analysis for prognosis highlights *CASZ1* and *CLEC12A* as protective factors and *RHOV*, *TNS4*, and *YWHAZ* as risk factors, consistent with previous studies. Notably, *S100A8* and *DNAH14*, previously unassociated, also emerged as significant prognostic risk factors. (Table 3, File S1, Figure S11A-N).

**Table 3:** The meta-analysis of mRNA expression differences and prognosis for Pre-RGs and Pro-RGs in LUAD

Gene	I² (%)	*P*	Observed SMD [95% CI]	Expression in Tumor	Prognostic HR [95% CI]	Prognostic Role
*S100A8*	89	4.6e-06	−1.45 [−2.03, −0.87]	Downregulated	1.21 [1.13, 1.29]	Risk Factor
*CLEC12A*	81	0.0089	−1.83 [−2.37, −1.30]	Downregulated	0.84 [0.77, 0.91]	Protective Factor
*CASZ1*	70	0.014	−1.26 [−1.64, −0.89]	Downregulated	0.87 [0.80, 0.95]	Protective Factor
*GPM6A*	94	1.7e-08	−3.63 [−4.63, −2.63]	Downregulated	-	-
*FEZ1*	86	6.1e-09	−1.96 [−2.51, −1.41]	Downregulated	-	-
*YWHAZ*	22	0.2200	1.28 [1.05, 1.51]	Upregulated	1.17 [1.05, 1.30]	Risk Factor
*OTX1*	95	6.2e-20	1.12 [0.10, 2.15]	Upregulated	-	-
*OCIAD2*	0	0.44	2.49 [2.27, 2.72]	Upregulated	-	-
*XPR1*	92	0.00011	1.99 [1.18, 2.80]	Upregulated	-	-
*SLC39A11*	78	0.01	2.01 [1.50, 2.51]	Upregulated	-	-
*DNAH14*	61	0.033	1.90 [1.53, 2.28]	Upregulated	1.13 [1.04, 1.24]	Risk Factor
*TNS4*	79	0.0014	0.82 [0.38, 1.26]	Upregulated	1.27 [1.16, 1.39]	Risk Factor
*XDH*	89	3.3e-09	1.08 [0.52, 1.64]	Upregulated	-	-
*RHOV*	0	0.28	1.52 [1.32, 1.73]	Upregulated	1.40 [1.26, 1.55]	Risk Factor

Abbreviation: Observed SMD [95% CI]: Standard Mean Difference in expression levels with 95% Confidence Interval; HR [95% CI]: Hazard Ratio for prognosis with 95% Confidence Interval

Additionally, pathway enrichment analysis of the 14 genes using Metascape (https://metascape.org/gp/index.html) [34] identified four pathways or biological processes: R-HSA-194,315: Signaling by Rho GTPases, GO:0098657: import into cell, GO:0009617: response to bacterium, and GO:0T098660: inorganic ion transmembrane transport (Supplementary material 2, Table S12). Further GSEA analysis based on single gene expression revealed significant enrichment in pathways such as olfactory signaling, formation of the cornified envelope, eukaryotic translation elongation, ribosome, olfactory transduction, tyrobp causal network in microglia, hdacs deacetylate histones, meiosis, keratinization, neuronal system, neuroactive ligand receptor interaction, hcmv late events, and other immune or cancer-related biological processes. Analysis of protein-protein interactions among the 14 genes using the STRING database did not reveal significant interactions (File S1, Figure S12A-O).

### Pre-RGs and Pro-RGs target drug or compound predictions

Using the online Comparative Toxicogenomics Database (CTD) [35], we investigated the Inference Scores of genes associated with LUAD. Multiple studies confirmed a strong association between *S100A8*, *GPM6A*, *YWHAZ*, *TNS4*, *XDH*, *SLC39A11*, *XPR1*, and LUAD (Supplementary material 2, Table S13). Additionally, we predicted target compounds for the 14 LUAD-associated genes, focusing on compounds that could modulate gene expression by either downregulating overexpressed genes or upregulating underexpressed ones. Compounds were ranked based on the number of target genes they affect. The compounds affecting ≥ 5 hub genes are: Abrine (C496492), Acetaminophen (D000082), Tretinoin (D014212), Cisplatin (D002945), Tobacco Smoke Pollution (D014028), Valproic Acid (D014635), and Trichostatin A (C012589) (details in Table 4). Based on clinical value and mechanistic evidence, the compounds are prioritized as follows: First-tier candidates: valproic acid, tretinoin, acetaminophen, and cisplatin (FDA-approved with well-defined mechanisms); Second-tier: trichostatin A (experimental HDAC inhibitor); Low-priority: abrine (limited data) and tobacco smoke (non-therapeutic). FDA-approved drugs are prioritized for potential repurposing. These compounds may aid in developing LUAD treatments and could serve as potential therapeutic candidates for LUAD.

**Table 4:** Compounds affecting expression of LUAD-Associated genes

Chemical ID	Chem.	Num.	Target Gene						
C496492	abrine	7	*S100A8* [38]	*CASZ1* [38]	*FEZ1* [38]	*OTX1* [38]	*XPR1* [38]	*SLC39A11*[38]	*TNS4* [38]
D000082	Acetaminophen	7	*CASZ1* [39]	*OTX1* [39]	*OCIAD2* [39, 40]	*XPR1* [41]	*SLC39A11* [39]	*RHOV* [41]	*XDH* [39, 40]
D014212	Tretinoin	7	*S100A8* [42]	*CLEC12A* [43]	*FEZ1* [44]	*YWHAZ* [45]	*SLC39A11* [43]	*DNAH14* [43]	*TNS4* [46]
D002945	Cisplatin	6	*CLEC12A* [47]	*FEZ1* [48]	*YWHAZ* [48]	*XPR1* [47]	*SLC39A11* [47]	*DNAH14* [48]	
D014028	Tobacco Smoke Pollution	6	*S100A8* [49–51]	*OTX1* [51]	*SLC39A11* [51, 52]	*TNS4* [52]	*RHOV* [51]	*XDH* [53]	
D014635	Valproic Acid	6	*S100A8* [54]	*CASZ1* [55–58]	*GPM6A* [54]	*FEZ1* [54, 55, 57, 59, 60]	*YWHAZ* [60]	*OTX1* [54]	
C012589	Trichostatin A	5	*CASZ1* [57, 61]	*GPM6A* [54]	*FEZ1* [57]	*OTX1* [57]	*XDH* [57]		

Abbreviation: *TSS* Transcription Start Sites, *KEGG* Kyoto Encyclopedia of Genes and Genomes

*GO* Gene Ontology, *BP* biological processes, *CC* Cellular Component, *MF* Molecular Function

Abbreviation: *DEGs* Differentially Expressed Genes, *DPGs* Differential peak genes, *CGs* Common Genes

### Non-invasive detection of LUAD from benign lung disease by plasma CfRNA

**Fig. 10:**
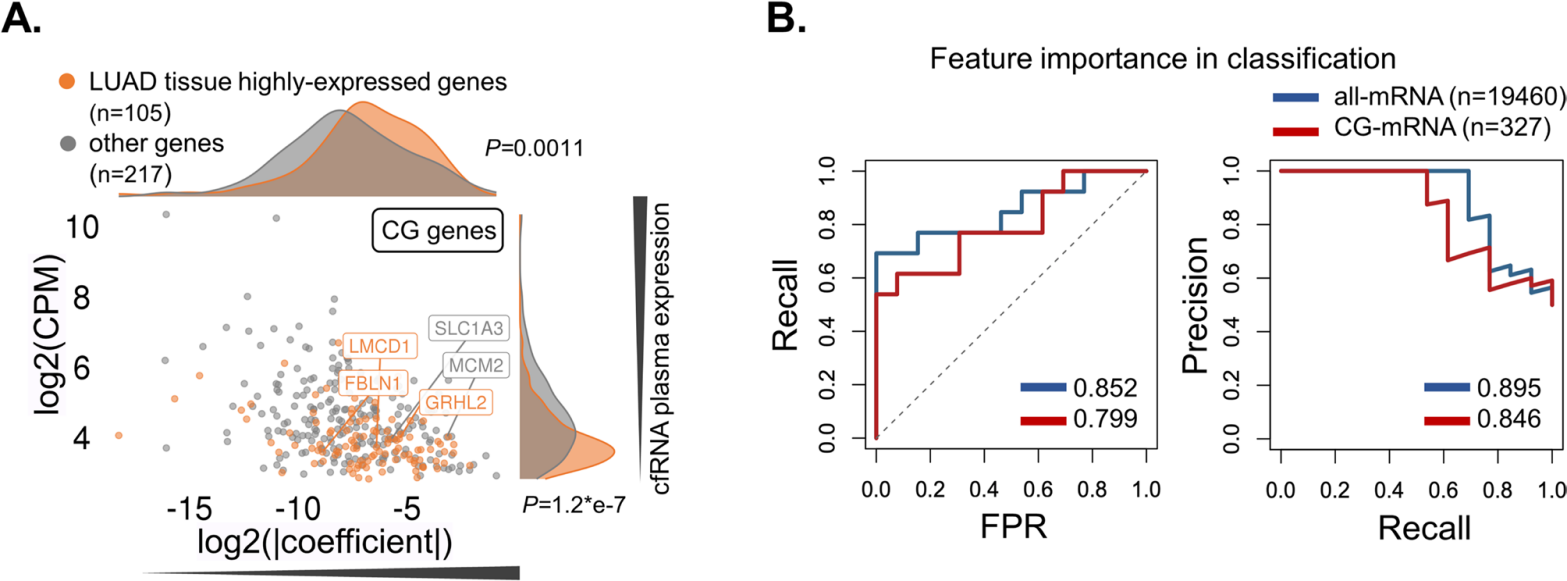
Cell-free CG transcripts as non-invasive plasma biomarker for LUAD non-invasive LUAD detection in in-house LUAD plasma cohort (A) Scatter plot showed the relation between peak expression and feature classification importance in the LUCA plasma cohort (in-house). X axis means log-transformed absolute coefficient in the LASSO models (higher value means more importance). Y axis means log-transformed CPM value. LUAD highly expressed (relative to primary blood cell) peaks were highlighted in orange. Consensus Genes (CG) were labeled in text. (B) AUROC and AUPR of all mRNA and CG mRNA in non-invasive classification of LUAD

We further tested CG’s application potential as a diagnostic approach in our in-house LUAD plasma cohort that includes CRC and pulmonary benign disease as normal control (NC) individuals. LASSO models were utilized for candidate protein-coding gene selection to classify plasma samples between LUAD and benign lung disease. We first explored the relationship between gene transcripts’ abundance in plasma and their classification importance in the All-mRNA models, and noticed many low abundant transcripts with greater importance, while those highly expressed in TCGA-LUAD tissue relative to primary blood cell showed significantly higher classification importance (*P* = 0.0011), which implied that cancer tissue-derived transcripts might contribute to the classification of LUAD from benign lung disease (Fig. 10A). For five CGs selected by All-mRNA models, three were annotated to be LUAD-highly expressed. Among them, *GRHL2* was reported to be associated with EMT status in lung cancer [36], and *FBLN1* was once reported as novel diagnostic biomarkers in both tissue and serum of lung cancer patients [37]. Generally, All-mRNA models using all coding genes as input achieved good classification performance (AUROC = 0.852, AUPR = 0.895), while those CG-mRNA models that used CGs solely showed a slightly lower but comparable performance (AUROC = 0.799, AUPR = 0.846) despite the significantly lower number of input genes (327 filtered CGs) for selection and modeling (Fig. 10B), which partially support the reliability of our previous identification of LUAD-associated genes from multi-omics data in tissue, and implied the application potential of these CGs as molecular biomarker for non-invasive detection of LUAD in plasma.

## Discussion

LUAD is a highly heterogeneous tumor influenced by various factors in its pathogenesis and prognosis. In this study, we constructed predictive and prognostic models for LUAD using ATAC-seq and RNA-seq data, and validated their predictive efficiency using external datasets. We also explored the associations of these models with immune infiltration, tumor microenvironment (TME), and immunotherapy response. Our comprehensive analysis revealed the expression patterns of predictive-related genes (Pre-RGs) and prognostic-related genes (Pro-RGs) in LUAD, and demonstrated their correlations with immune infiltration, TME, and patient prognosis, providing new insights into LUAD pathogenesis and treatment.

Firstly, ATAC-seq analysis showed that open chromatin regions were widely distributed across promoter and distal regulatory elements, which play crucial roles in LUAD progression. Functional enrichment analysis indicated significant involvement in cancer-related signaling pathways, cellular morphogenesis, intracellular transport, kinase activity regulation, and infection- and cell cycle-related metabolic processes. Through random forest and LASSO analysis, we identified nine key Pre-RGs (*S100A8*, *GPM6A*, *FEZ1*, *OTX1*, *DNAH14*, *XDH*, *XPR1*, *SLC39A11*, *OCIAD2*) and constructed a neural network-based diagnostic model that demonstrated excellent diagnostic performance in both training and validation cohorts (AUC = 1.000 and 0.978). These results strongly suggest the potential of these genes as diagnostic biomarkers for LUAD. For prognostic analysis, we identified five Pro-RGs (*TNS4*, *RHOV*, *YWHAZ*, *CLEC12A*, *CASZ1*) through univariate Cox analysis, Kaplan-Meier analysis, LASSO dimensionality reduction, and multivariate Cox regression. Based on these genes, we developed a risk model that stratified patients into high- and low-risk groups. High-risk patients exhibited lower TME scores, reduced immune cell infiltration, and significant enrichment in cell cycle and DNA replication pathways, suggesting poor immune responses and reduced immunotherapy sensitivity. In contrast, low-risk patients showed enrichment in immune- and adhesion-related pathways, stronger immune-related functions, and greater sensitivity to immunotherapy, consistent with better overall survival. The risk score also showed a moderate but significant positive correlation with cancer stem cell index (*R* = 0.29), indicating its potential to reflect tumor stemness and aggressiveness. Furthermore, single-cell RNA-seq analysis revealed the expression patterns of these genes across different cell subpopulations, confirming their biological relevance at the cellular level. These findings provide important evidence for the clinical application of these genes as prognostic biomarkers and potential therapeutic targets. Importantly, most immune checkpoint genes were more highly expressed in the low-risk group, suggesting better potential responses to immune checkpoint inhibitor therapy. Moreover, patients in the low-risk group were predicted to be more sensitive to multiple chemotherapeutic and targeted agents, further supporting the clinical significance of this stratification. Meta-analysis and pathway enrichment confirmed that these genes participate in critical biological processes, including Rho GTPase signaling, cell import, response to bacterium, and ion transport. While no significant PPI network was identified among these genes, their consistent differential expression patterns and associations with prognosis emphasize their individual importance.

Despite the comprehensive bioinformatics analyses, our study lacks in vitro and in vivo functional validation for key genes, such as *RHOV* and *CLEC12A*. Functional experiments (e.g., gene knockout, overexpression, or mechanistic assays) will be necessary to confirm the biological roles and mechanistic contributions of these genes to LUAD progression and treatment response. We acknowledge this as a limitation of the current study and propose it as a future research direction. Moreover, while single-cell analysis provides valuable insights into the cellular context of these genes, further validation using larger and independent single-cell cohorts will strengthen our conclusions regarding cell-type specificity and cell–cell communication patterns. Integration of Pre-RGs and Pro-RGs into clinical workflows may offer valuable molecular insights beyond traditional histopathological and imaging assessments, enabling more precise treatment strategies for LUAD patients. Low-risk patients identified by our prognostic model, characterized by higher immune infiltration and robust immune signatures, are more likely to benefit from immune checkpoint inhibitors. In contrast, high-risk patients, exhibiting enhanced cell cycle activity and reduced immune cell infiltration, may respond better to combination regimens incorporating targeted therapies (e.g., CDK inhibitors) or chemotherapy to overcome resistance and suppress tumor progression. Moreover, evaluating the expression patterns of these key genes could help predict sensitivity to specific targeted agents, allowing for individualized treatment plans that optimize efficacy and minimize toxicity. Incorporating these molecular signatures into routine clinical practice can facilitate patient stratification, inform dynamic treatment adjustments, and ultimately improve survival and quality of life. This approach also lays the groundwork for future clinical trials to validate gene-based precision strategies and refine therapeutic algorithms in LUAD.

In conclusion, this study elucidates new pathophysiological mechanisms in LUAD and proposes promising diagnostic and prognostic biomarkers and therapeutic targets for future clinical applications.

## Supplementary Information

Supplementary material 1.

Supplementary material 2.

## Data Availability

The datasets analyzed in this study are publicly available in the TCGA (https://portal.gdc.cancer.gov/) and GEO (https://www.ncbi.nlm.nih.gov/geo/) repositories. The relevant accession numbers are provided within the manuscript.Additional datasets generated or analyzed during this study are not publicly available due to institutional data privacy policies. However, they are available from the corresponding author upon reasonable request and with the necessary institutional approvals. The cfRNA dataset has been deposited in the GEO database under accession number GSE278695 (secure token: mfcxiuimnxkjdal). The code used in this study is publicly available at [https://github.com/LuZDPUMC/LUAD-paper-code.git](https:/github.com/LuZDPUMC/LUAD-paper-code.git).

## Abbreviations

LUAD: Lung Adenocarcinoma
ATAC-seq: Assay for Transposase-Accessible Chromatin using sequencing
TSS: Transcription Start Sites
K-M: Kaplan-Meier method
LASSO: Least Absolute Shrinkage and Selection Operator
OS: Overall Survival
AUC: Area Under the Curve
CI: Confidence Interval
GEO: Gene Expression Omnibus
IFNG: Interferon Gamma
CD274: Programmed Cell Death 1 Ligand 1, PD-L1
MDSC: Myeloid-Derived Suppressor Cells
TAM M2: M2-type Tumor-Associated Macrophages
*XDH*: Xanthine dehydrogenase
*SLC39A11*: Solute carrier family 39 member 11
*GPM6A*: Glycoprotein M6A
*XPR1*: Xenotropic and polytropic retrovirus receptor 1
*FEZ1*: Fasciculation and elongation protein zeta 1
*OTX1*: Orthodenticle homeobox 1
*S100A8*: S100 calcium binding protein A8
*DNAH14*: Dynein axonemal heavy chain 14
*OCIAD2*: OCIA domain containing 2
*TNS4*: Tensin 4
*RHOV*: Ras homolog family member V
*YWHAZ*: Tyrosine 3-monooxygenase/tryptophan 5-monooxygenase activation protein zeta
*CLEC12A*: C-type lectin domain family 12 member A
*CASZ1*: Castor zinc finger 1
TCIA: The Cancer Immunome Database
