# Supplementary material for: Multi-omics data-based modeling reveals tumorigenesis- and prognosis-associated genes with clinical potential in lung adenocarcinoma
Source: BMC Cancer. 2025 Nov 10;25:1743. doi: 10.1186/s12885-025-14943-x (PMC12604227; doi:10.1186/s12885-025-14943-x)
Supplement: Supplementary file 2 — Supplementary material 2. [file 12885_2025_14943_MOESM2_ESM.pdf]

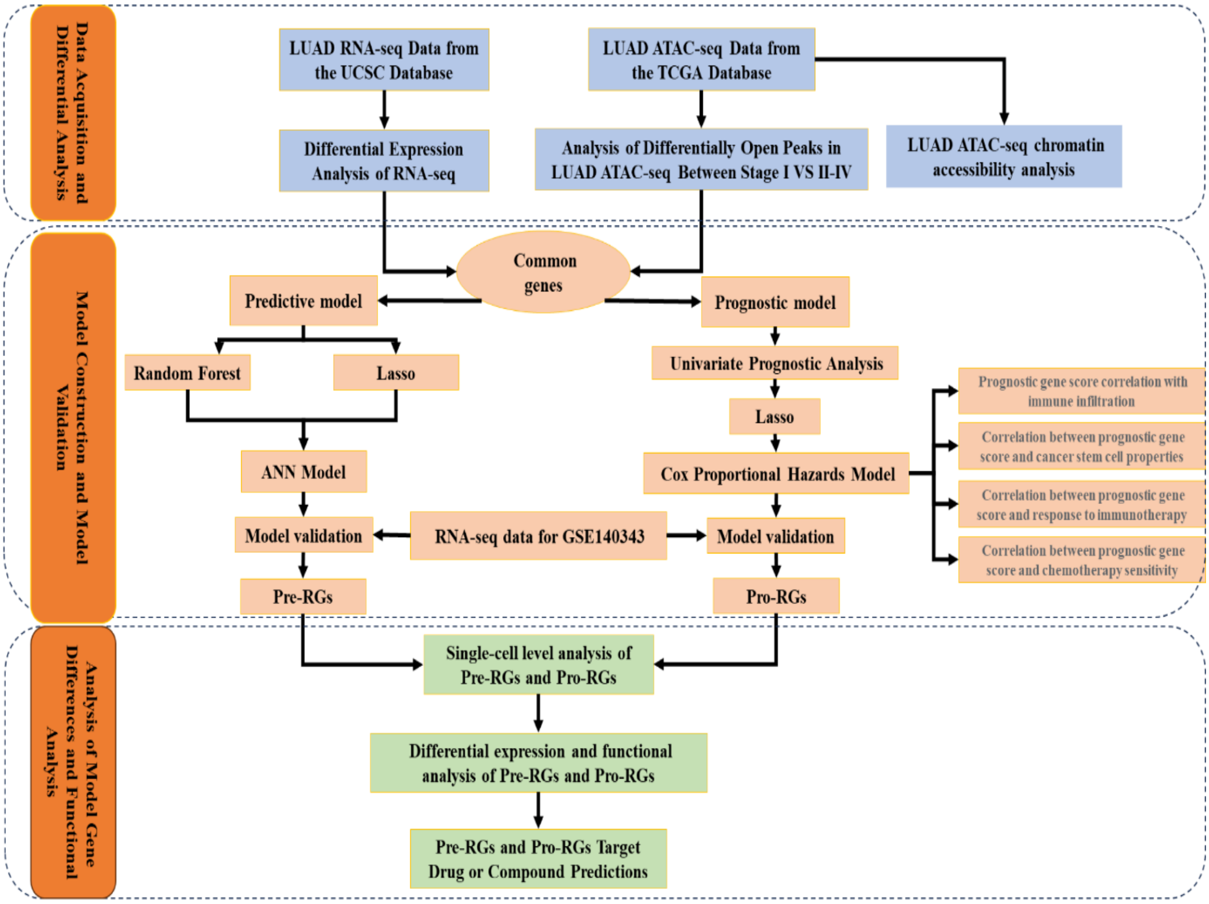

Figure S1. Flowchart of this study.

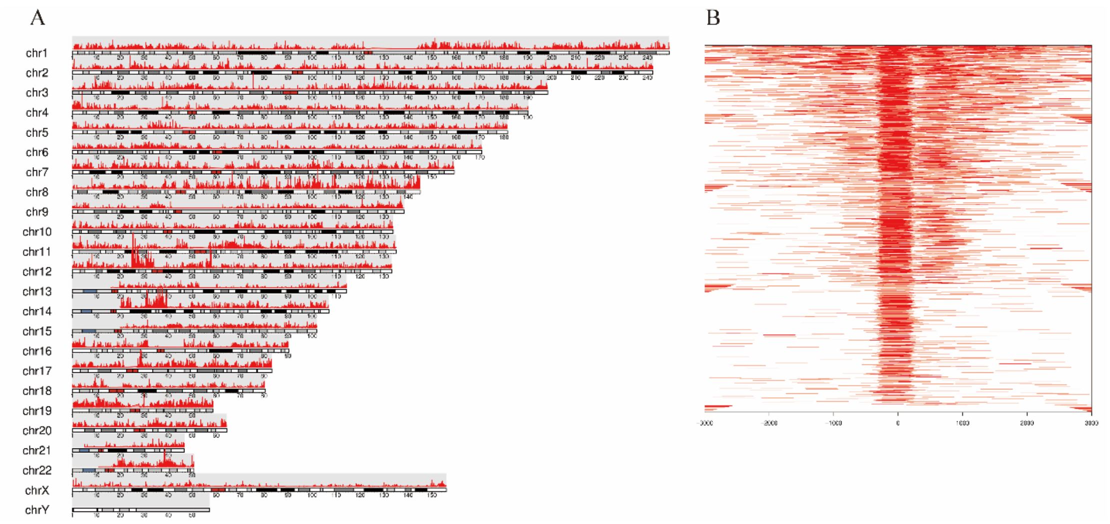

Figure S2. A) Covered length of ATAC-seq peaks of LUAD. The abscissa represents the chromosome position, and the red band represents the standardized value of peaks. B) Heat map of distance from peaks to transcription initiation sites in ATAC-seq data of LUAD.

Abbreviation: ATAC-seq: Assay for transposase-accessible chromatin using sequencing; LUAD: Lung adenocarcinoma; Chr: Chromosome.

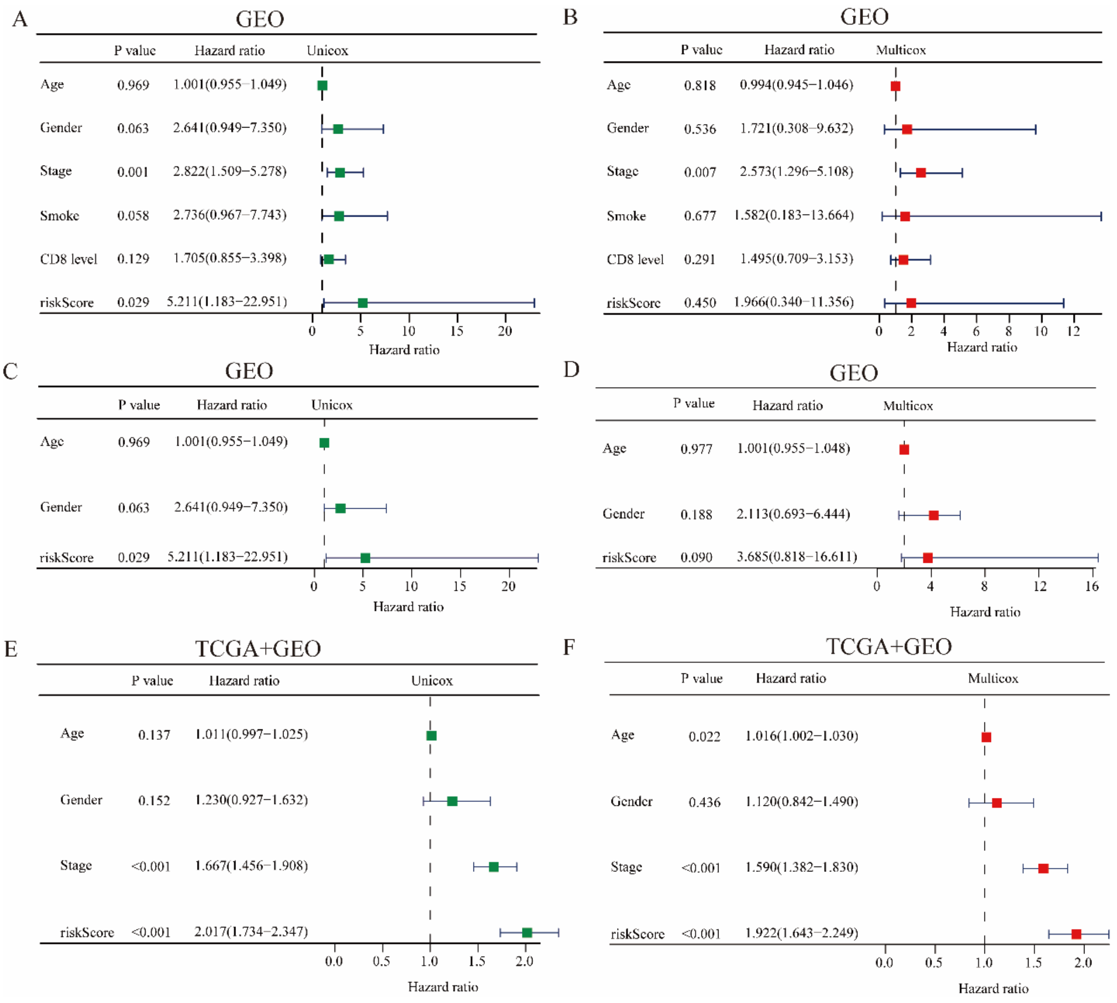

Figure S3. Analysis of Risk Score as an Independent Prognostic Factor for OS

A-B) Univariate (A) and multivariate (B) Cox regression analyses of independent prognostic factors based on GEO validation data. C-D) Univariate (C) and multivariate (D) Cox regression analyses of independent prognostic factors based on GEO data after removing factors potentially collinear with risk scores. E-F) Univariate (E) and multivariate (F) Cox regression analyses of independent prognostic factors based on the combined TCGA and GEO data.

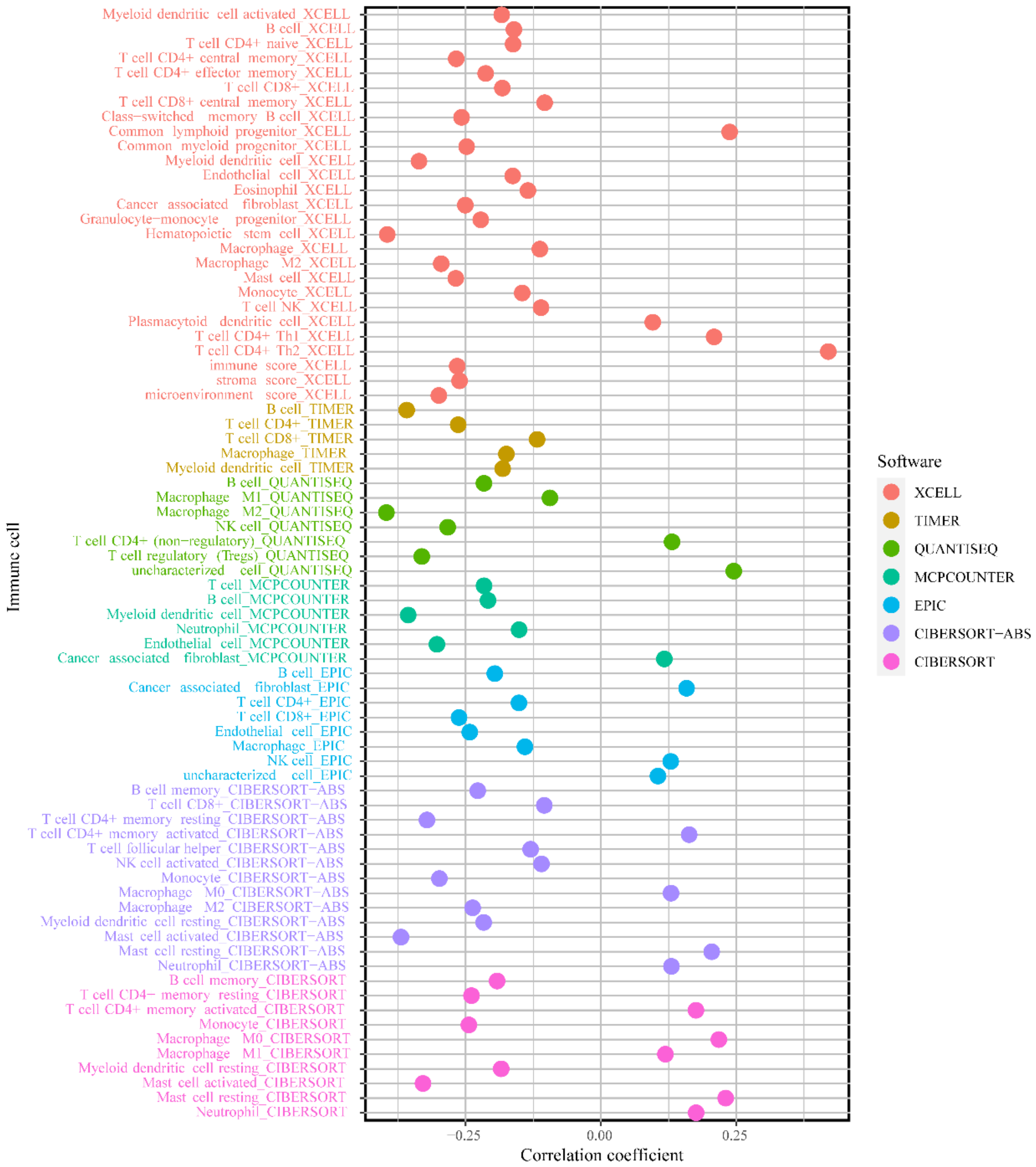

Figure S4. Correlation Analysis Between Immune Infiltration and Risk Scores.

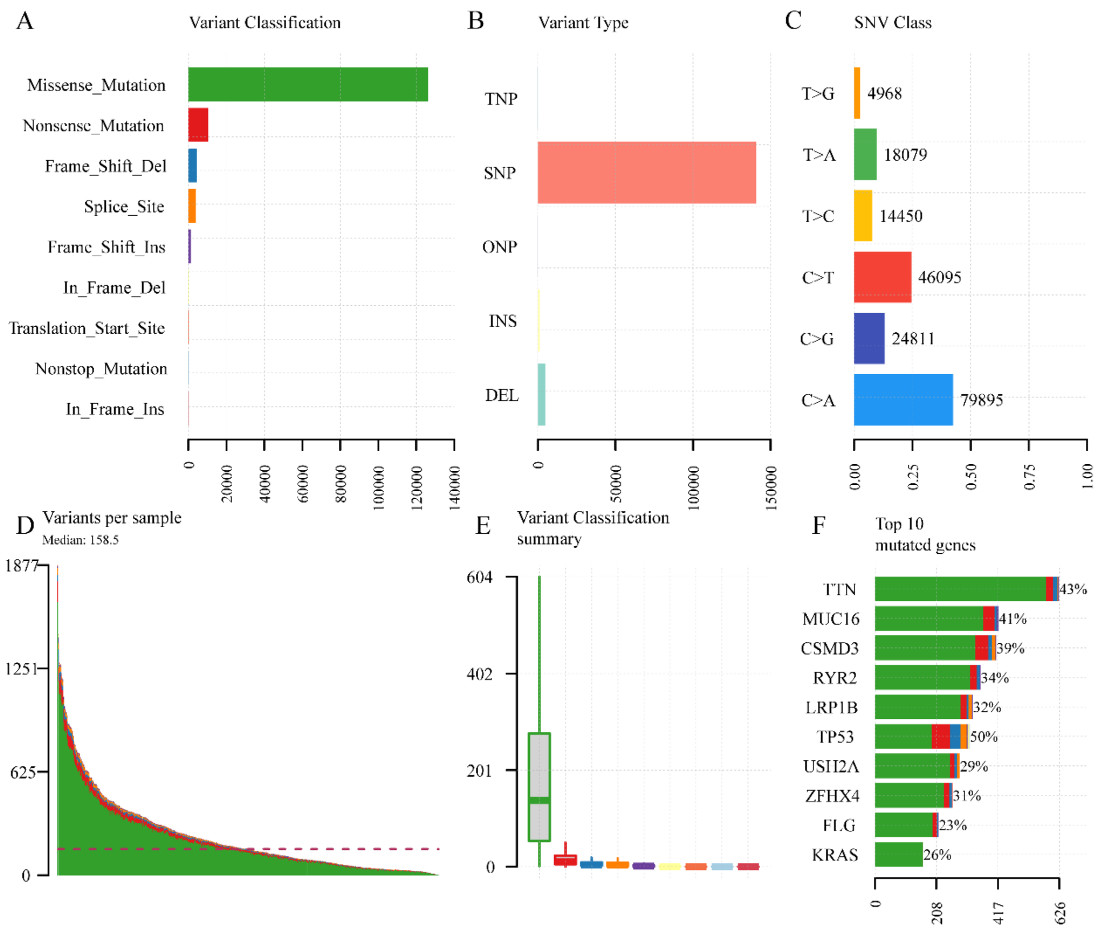

Figure S5. Summary of Tumor Mutation Profile in TCGA LUAD Patients.

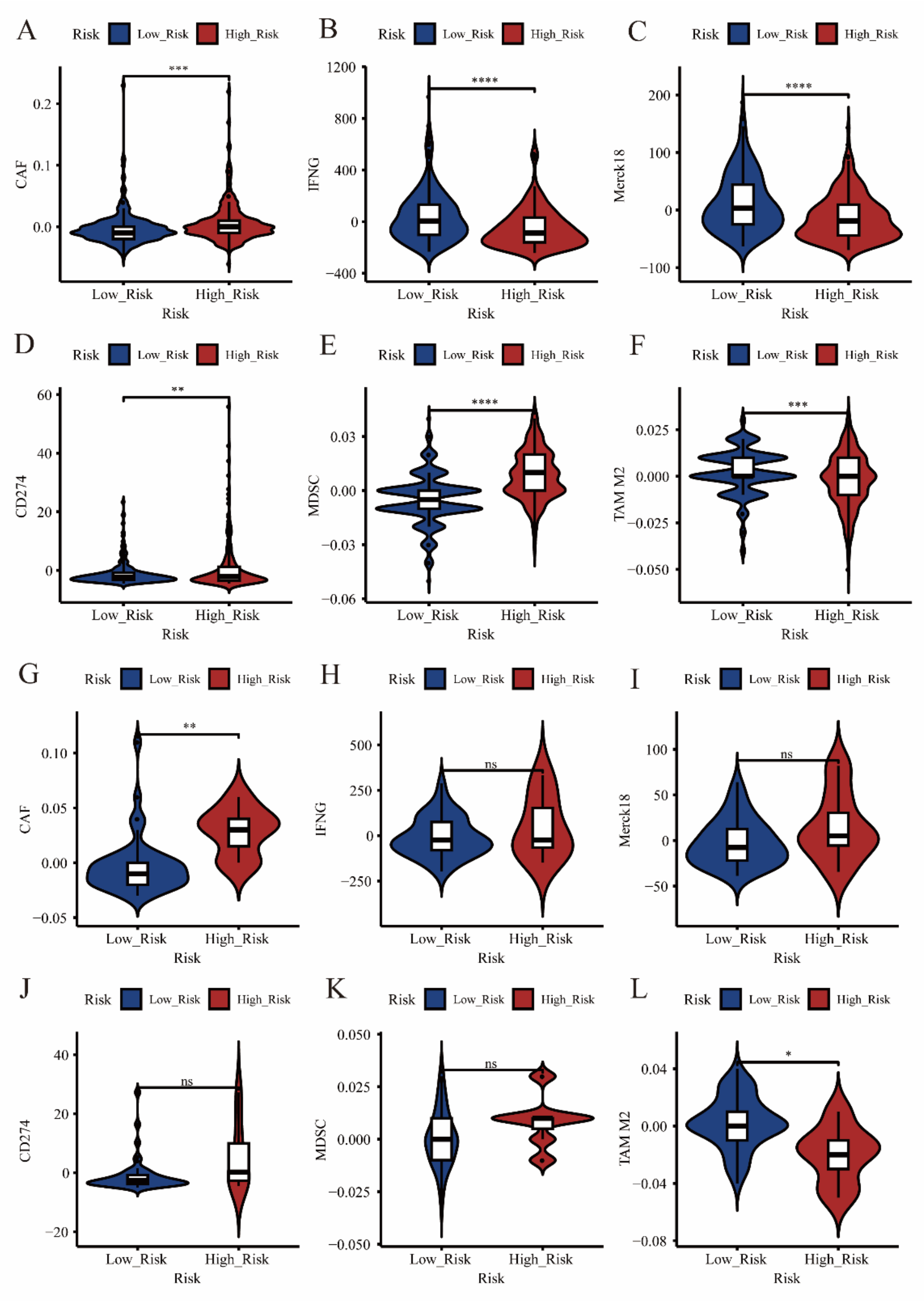

Figure S6. Immunotherapy-Related Biomarkers in Relation to Risk Groups Based on TIDE Analysis Data. Mean values of CAF, IFNG, Merck18, CD274, MDSC, CAF, and TAM M2 are compared between low-risk and high-risk groups in the training set (TCGA) (A-F) and validation set (GEO) (G-L).

Abbreviation: CAF: Cancer-Associated Fibroblasts; IFNG: Interferon Gamma; Merck18: Marker of Response to Anti-PD1 Therapy (Merck18); CD274: Programmed Cell Death 1 Ligand 1 (PD-L1); MDSC: Myeloid-Derived Suppressor Cells; TAM M2: M2-type Tumor-Associated Macrophages.

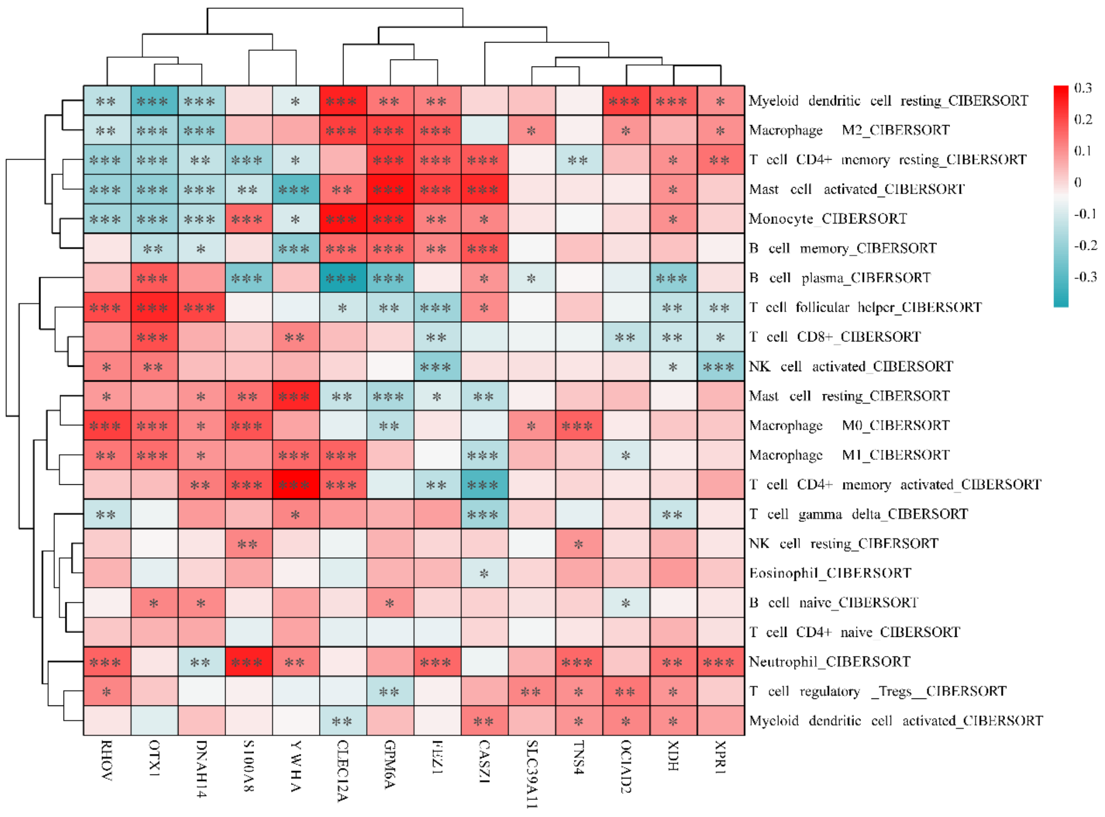

Figure S7. Correlation Between 14 Genes and Immune Cells.

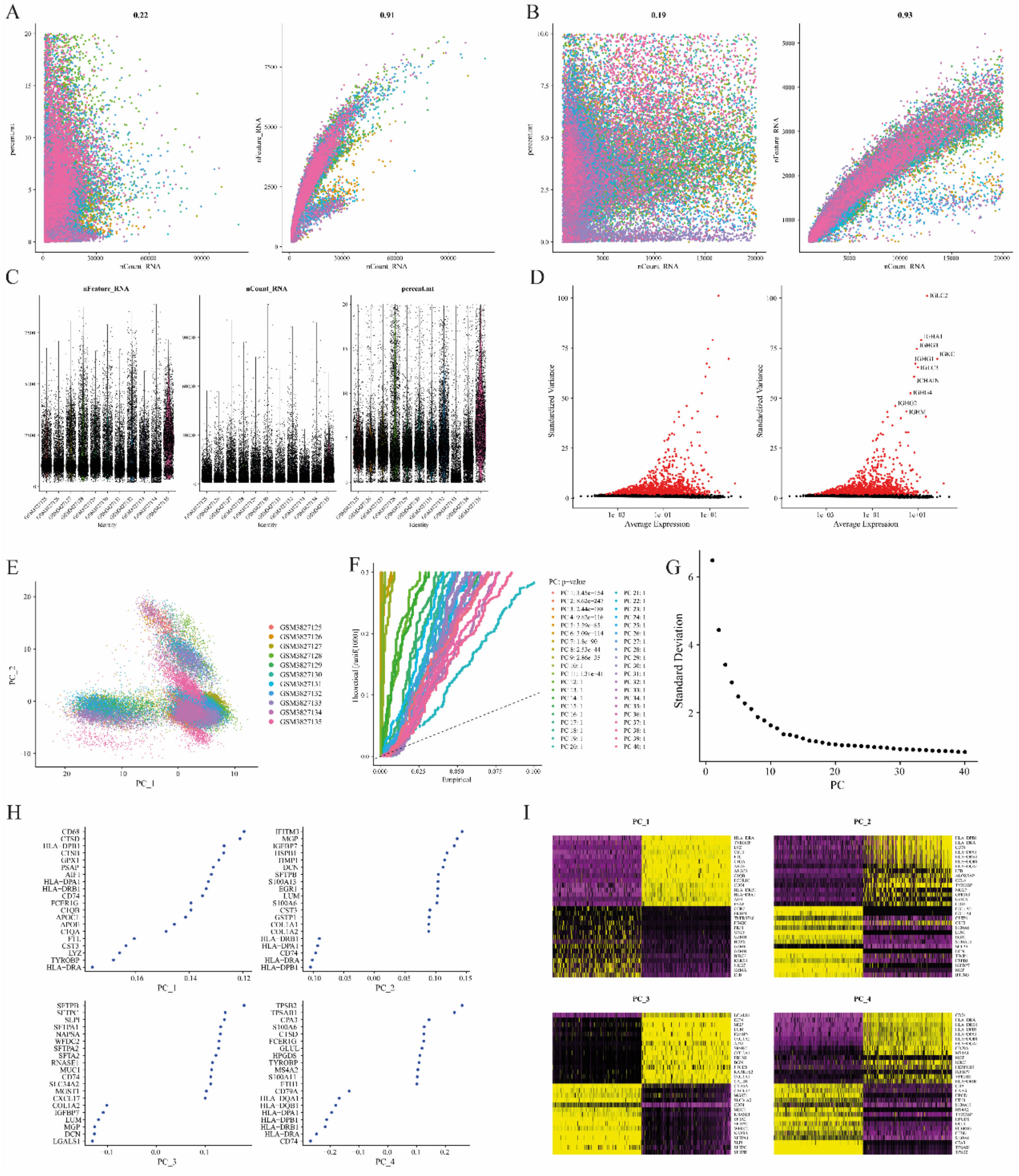

Figure S8. Standard Workflow for Single-Cell Analysis. Visualize the relationship between nFeature_RNA, nCount_RNA, and percent.mt before (A) and after (B) quality control using scatter plots; (C) Visualize QC metrics as a violin plot; (D) Identify highly variable genes and display the top ten highly variable genes; (E) Scatter plot of single-cell RNA sequencing data across PCA components 1 and 2, showing cell distribution and heterogeneity. Use JackStrawPlot (F) and Elbow plot (G) to determine the dimensionality of the dataset. Bubble plot (H) and heatmap (I) visualize the top four features represented by PCA rankings.

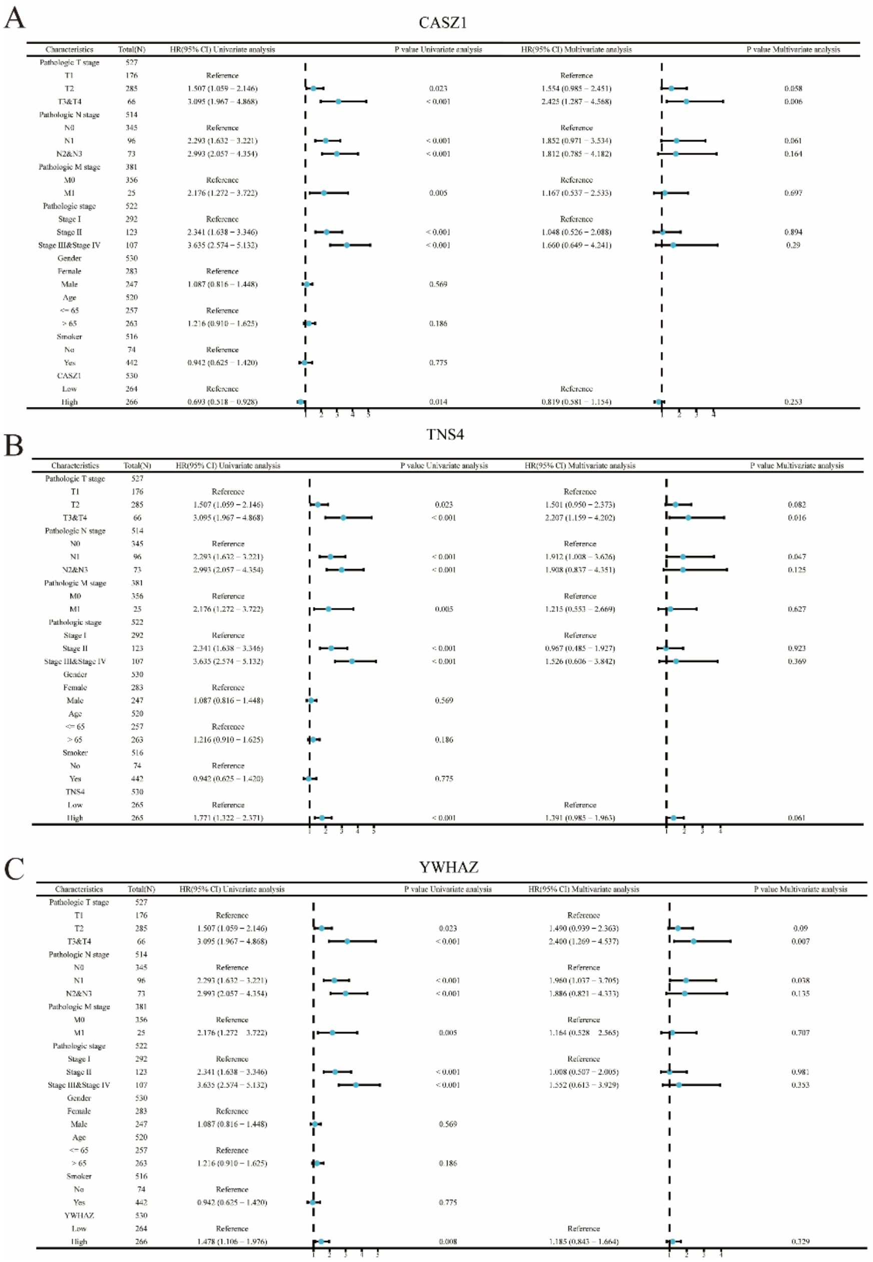

Figure S9. Independent Prognostic Analysis of Pro-RGs in LUAD: (A) CASZ1, (B) TNS4, (C) YWHAZ.

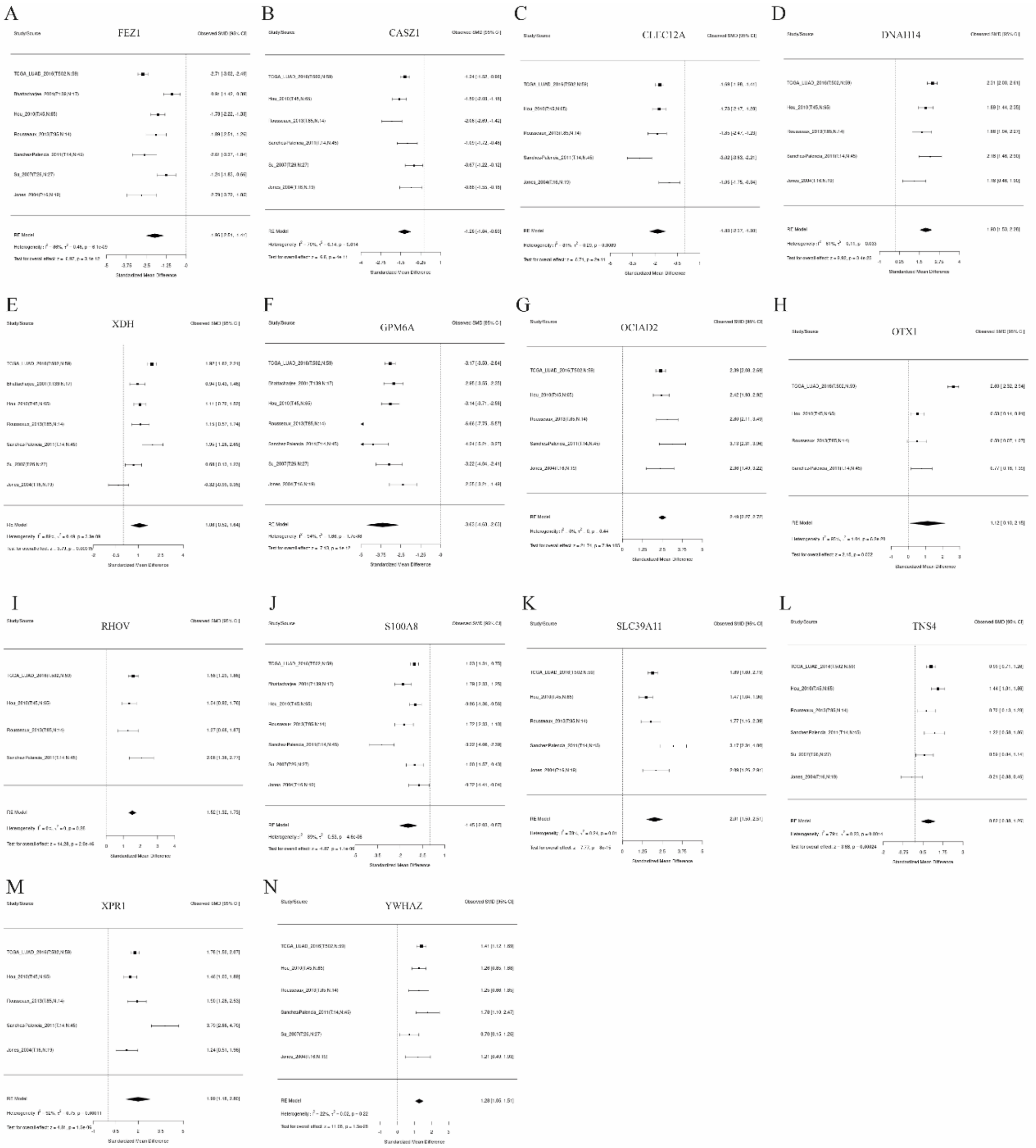

Figure S10. Meta-Analysis of mRNA Expression of Pre-RGs and Pro-RGs in LUAD.

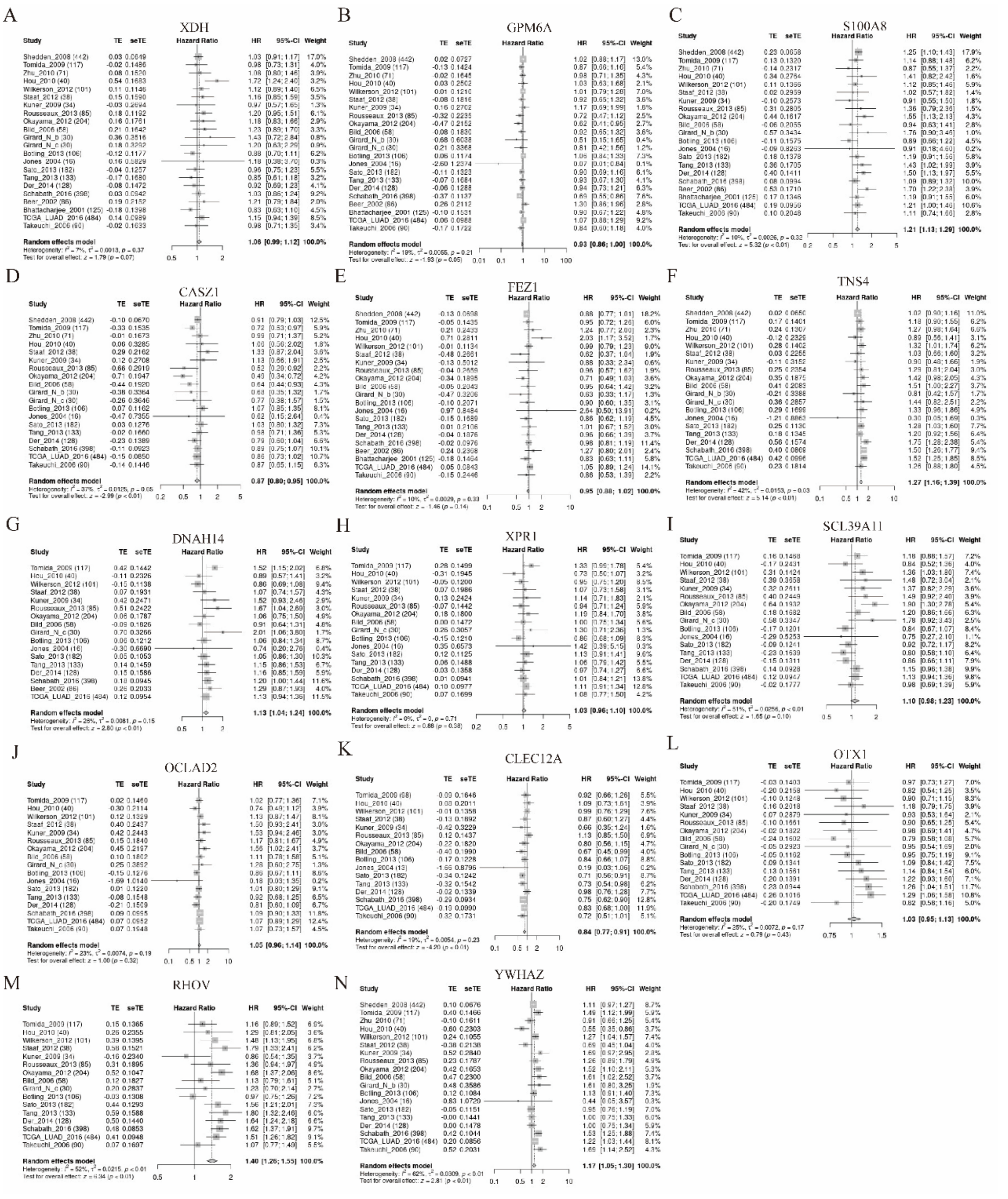

Figure S11. Meta-Analysis of Prognostic Factors for Pre-RGs and Pro-RGs in LUAD.

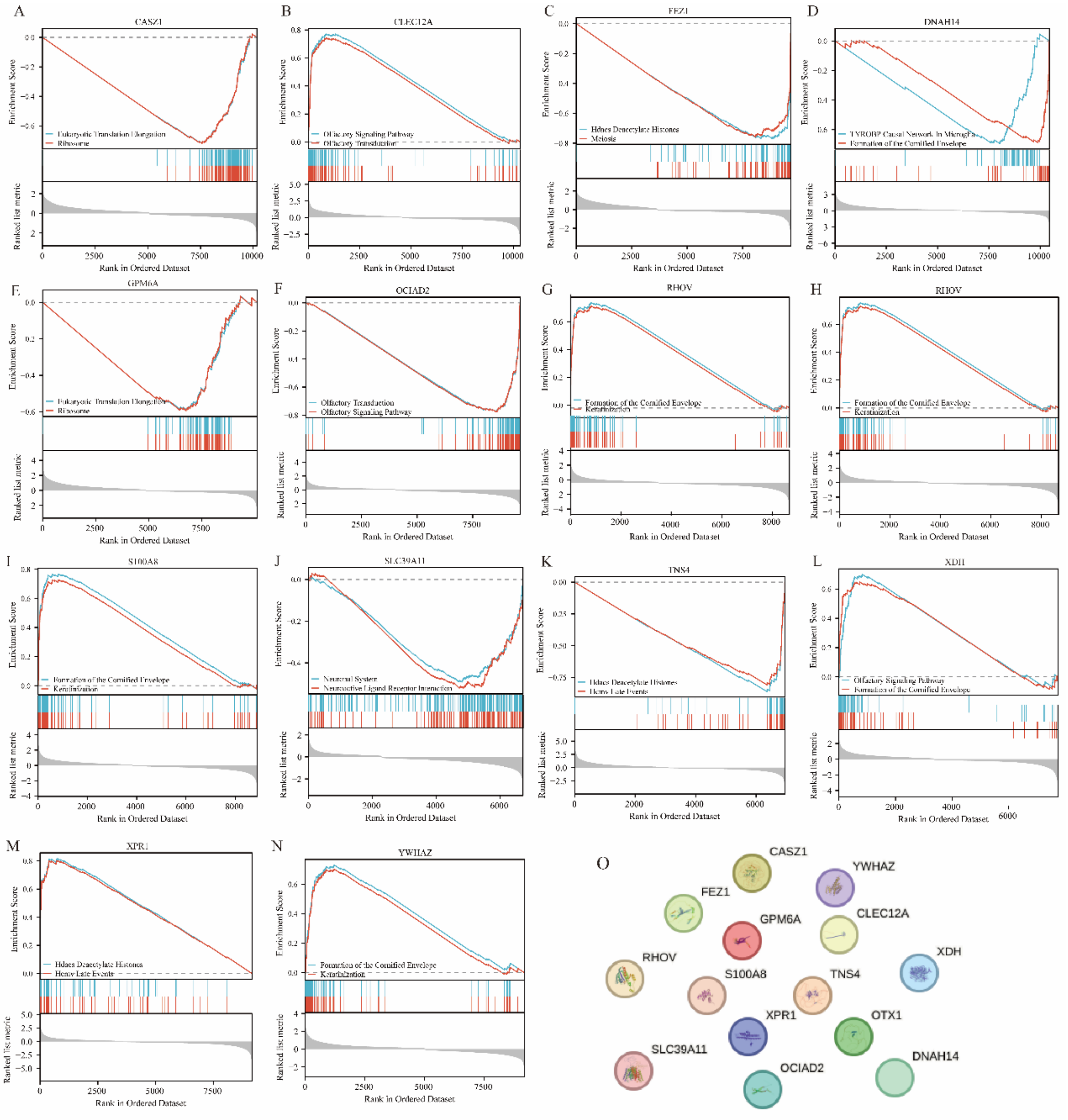

Figure S12. Functional Analysis and Protein-Protein Interactions of 14 Genes in LUAD. GSEA Analysis of 14 Genes (A-N) and Protein-Protein Interaction Analysis (O).
